# Adaptor protein RapZ activates endoribonuclease RNase E by protein–protein interaction to cleave a small regulatory RNA

**DOI:** 10.1261/rna.074047.119

**Published:** 2020-09

**Authors:** Svetlana Durica-Mitic, Yvonne Göpel, Fabian Amman, Boris Görke

**Affiliations:** 1 Department of Microbiology, Immunobiology and Genetics, Max Perutz Labs, University of Vienna, Vienna Biocenter (VBC), 1030 Vienna, Austria; 2 Center for Anatomy and Cell Biology, Medical University of Vienna, 1090 Vienna, Austria; 3 Institute of Theoretical Biochemistry, University of Vienna, 1090 Vienna, Austria

**Keywords:** RNA-binding protein, adaptor protein RapZ, endoribonuclease RNase E, regulated RNA degradation, small RNA GlmZ

## Abstract

In *Escherichia coli*, endoribonuclease RNase E initiates degradation of many RNAs and represents a hub for post-transcriptional regulation. The tetrameric adaptor protein RapZ targets the small regulatory RNA GlmZ to degradation by RNase E. RapZ binds GlmZ through a domain located at the carboxyl terminus and interacts with RNase E, promoting GlmZ cleavage in the base-pairing region. When necessary, cleavage of GlmZ is counteracted by the homologous small RNA GlmY, which sequesters RapZ through molecular mimicry. In the current study, we addressed the molecular mechanism employed by RapZ. We show that RapZ mutants impaired in RNA-binding but proficient in binding RNase E are able to stimulate GlmZ cleavage in vivo and in vitro when provided at increased concentrations. In contrast, a truncated RapZ variant retaining RNA-binding activity but incapable of contacting RNase E lacks this activity. In agreement, we find that tetrameric RapZ binds the likewise tetrameric RNase E through direct interaction with its large globular domain within the catalytic amino terminus, independent of RNA. Although RapZ stimulates cleavage of at least one non-cognate RNA by RNase E in vitro, its activity is restricted to GlmZ in vivo as revealed by RNA sequencing, suggesting that certain features within the RNA substrate are also required for cleavage. In conclusion, RapZ boosts RNase E activity through interaction with its catalytic domain, which represents a novel mechanism of RNase E activation. In contrast, RNA-binding has a recruiting role, increasing the likelihood that productive RapZ/GlmZ/RNase E complexes form.

## INTRODUCTION

In diverse bacteria, endoribonuclease RNase E usually initiates decay of bulk RNA (Ono and Kuwano 1979; Mackie 2013b; Ait-Bara and Carpousis 2015; Bandyra and Luisi 2018), participates in RNA processing and is also involved in post-transcriptional gene regulation mediated by small regulatory RNAs (sRNAs) (Vogel and Luisi 2011; Waters et al. 2017). RNase E contributes to sRNA biogenesis, maturation as well as decay (Göpel et al. 2013; Miyakoshi et al. 2015; Chao et al. 2017). Some sRNAs recruit RNase E to degrade the target RNA, while others have protective roles by occlusion of RNase E cleavage sites upon base-pairing (Morita et al. 2005; McCullen et al. 2010; Fröhlich et al. 2013; Baek et al. 2019).

**FIGURE 1.**
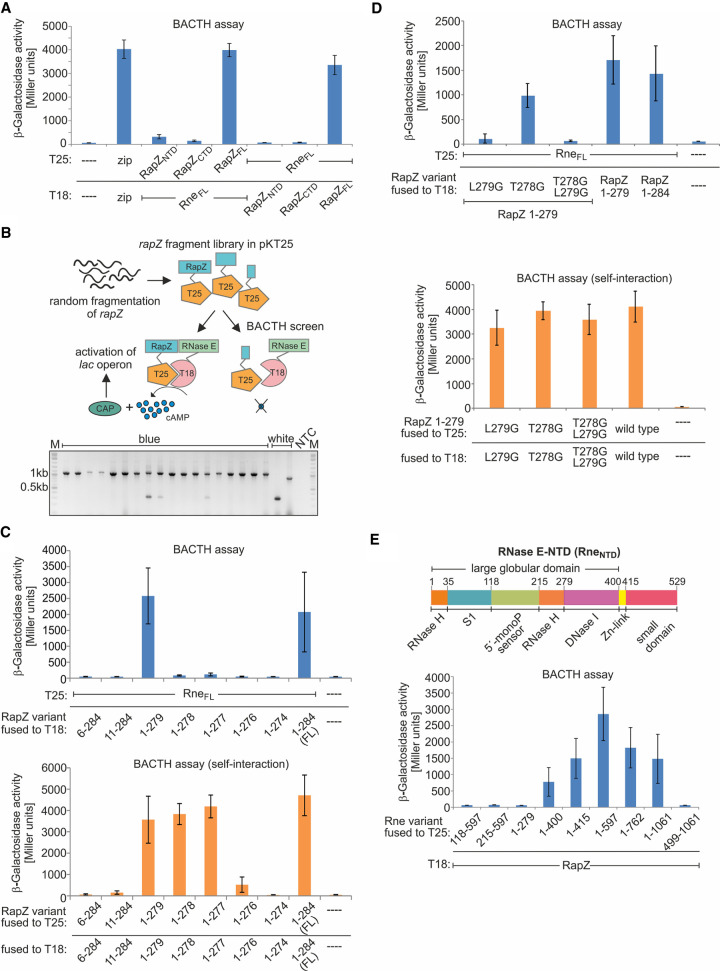
Mapping the interaction surfaces in RapZ and RNase E. (*A*) BACTH assay addressing interaction of the two globular domains of RapZ with Rne_FL_. Used plasmids were: pKT25, pKT25-zip, pSD9, pSD10, pBGG348, pYG100 (encoding T25 or T25-fusions) and pUT18C, pUT18C-zip, pYG99, pSD11, pSD12, pBGG349 (encoding T18 or T18-fusions). The positive control is provided by plasmids pKT25-zip and pUT18-zip, which encode the leucine zipper of yeast transcription factor Gcn4 fused to the T25 and T18 domain, respectively (column 2). (*B*) *Top*: strategy of the BACTH screen for RapZ truncations retaining interaction with RNase E. Randomly sized *rapZ* DNA fragments were shotgun cloned into pKT25 and the resulting library was screened in BTH101 for interaction with T18-Rne_FL_. The *rapZ* inserts of blue as well as white recombinants were PCR-amplified and fragment sizes were determined by agarose gel electrophoresis (*bottom*). NTC denotes no template control. See Supplemental Figure S1A for analysis of additional recombinants. (*C*) *Top*: BACTH analysis of interaction between defined RapZ truncations (fused to T18) and T25-Rne_FL_ (encoded on plasmid pYG100). The following T18 encoding plasmids were used: pSD111, pSD112, pSD113, pSD140, pSD141, pSD133, pSD114, pBGG349, pUT18C. *Bottom*: self-interaction properties of the RapZ truncations. The T18-constructs from *top* were combined with plasmids encoding the same RapZ variants fused to T25 (listed in Supplemental Table S10). (*D*) BACTH analysis addressing the roles of residues Leu279 and Thr278 in RapZ_1–279_ for interaction with Rne_FL_ (*top*) and self-oligomerization (*bottom*). The T18-RapZ_1–279_ variants carrying the indicated substitutions of residues 279 and/or 278 were encoded on plasmids pSD192, pSD193 and pSD194, respectively. Plasmid pBGG349 encoding T18-RapZ_FL_ was included for comparison. To test for interaction with RNase E, cells additionally carried plasmid pYG100 encoding T25-Rne_FL_. To test for self-interaction, cells additionally carried the following plasmids encoding the same RapZ variant but fused to T25: pSD197, pSD198, and pSD199. A strain producing the non-mutated T18- and T25-RapZ_1–279_ variants from plasmids pSD113 and pSD118 was included for comparison. A strain carrying the empty BACTH plasmids pKT25 and pUT18C served as negative control. (*E*) *Top*: schematic representation of the Rne_NTD_ domain organization. *Bottom*: BACTH analysis of interaction between T18-RapZ (encoded on plasmid pBGG349) with truncations of RNase E fused to T25. The various T25-Rne fusions were encoded on plasmids pSD7, pSD8, pSD6, pSD5, pSD3, pYG101, pSD2, pYG100, pYG102. Data information: β-galactosidase activities are presented as mean ± SD. (*A,C*,*E*) *n* ≥ 3; (*D*) *n* ≥ 2. Two-tailed Student's *t*-tests were performed to assess whether two data sets are significantly different. The calculated *P*-values are reported in the source data file (Supplemental Table S3).

RNase E is tetrameric and composed of a dimer of dimers (Callaghan et al. 2005). The catalytic activity resides in the amino-terminal domain (Rne_NTD_; aa 1–529) (McDowall and Cohen 1996; Callaghan et al. 2005), which consists of a large globular domain (aa 1–400), a Zn-link (aa 400–415) stabilizing dimer formation and a small folded domain (aa 415–529) mediating tetramerization (Fig. 1E, top; Bandyra and Luisi 2018). The large globular domain is a composite of subdomains comprising the RNase H-like domain, the S1 domain contributing to RNA-binding, the 5′ monophosphate sensing pocket, and the DNase I domain hydrolyzing RNA (Callaghan et al. 2005). The carboxy-terminal domain of RNase E (Rne_CTD_; aa 530–1061) is intrinsically disordered and contains binding sites for RNA and proteins as well as a membrane attachment site (Callaghan et al. 2004; Tsai et al. 2012; Strahl et al. 2015). RNA helicase B, enzyme enolase and exoribonuclease PNPase bind to the Rne_CTD_, collectively forming the degradosome (Miczak et al. 1996; Leroy et al. 2002). These are the main components of the core degradosome, but its composition may change depending on the growth conditions.

RNase E cleaves single-stranded RNA at AU-rich sites (McDowall et al. 1994; Chao et al. 2017). Two pathways for substrate recognition by RNase E are known. The 5′ end-dependent mode relies on a 5′ terminal monophosphate group in the RNA, which interacts with a sensing pocket in Rne_NTD_. This allosteric interaction converts RNase E into a closed conformation enabling appropriate orientation of the substrate and boosting enzyme activity (Callaghan et al. 2005; Garrey et al. 2009; Bandyra et al. 2018). To enter this pathway, primary transcripts require conversion to 5′ monophosphorylated variants in a two-step process involving pyrophosphohydrolase RppH (Deana et al. 2008; Luciano et al. 2017). A second group of substrates comprises transcripts that are directly cleaved at internal sites, regardless of the 5′ end phosphorylation status (Kime et al. 2010; Clarke et al. 2014). The direct entry mode may involve recognition of a duplex region in the substrate, but single-stranded regions of appropriate length providing handholds for RNase E also appear to be important (Kime et al. 2014; Bandyra et al. 2018; Updegrove et al. 2019).

Activity of RNase E can be altered through recruitment of alternative interaction partners. For instance, proteins RraA and RraB bind the Rne_CTD_ at distinct sites and inhibit degradosome activity by altering its composition (Lee et al. 2003; Gao et al. 2006; Gorna et al. 2010). RNase E is also targeted by phage proteins, which inhibit or stimulate its activity to promote infection (Qi et al. 2015; Van den Bossche et al. 2016). The latter proteins affect RNase E activity globally. However, two proteins are known to target specific transcripts, that is, sRNAs to degradation by RNase E. CsrD triggers degradation of sRNAs CsrB/CsrC in response to carbon source availability (Leng et al. 2016). The other example is RapZ, an adaptor protein that is essential for cleavage of sRNA GlmZ by RNase E in *E. coli*. Aided by Hfq, GlmZ activates translation of the *glmS* mRNA by base-pairing (Kalamorz et al. 2007; Urban and Vogel 2008). Enzyme GlmS synthesizes glucosamine-6-phosphate (GlcN6P), a key metabolite required for cell envelope biosynthesis. When GlcN6P levels are ample, GlmZ is inactivated by RNase E-catalyzed cleavage in the base-pairing region, a process strictly dependent on RapZ (Göpel et al. 2013). RapZ binds GlmZ and also RNase E, notably by interaction with the catalytic domain. GlmZ consists of three stem–loop structures, the central of which is critical for RapZ binding and decisive for cleavage that occurs at a fixed distance in the single-stranded region downstream from this structure (Göpel et al. 2013, 2016). Cleavage is counteracted by the homologous sRNA GlmY, which sequesters RapZ through molecular mimicry upon GlcN6P starvation. Recent results have shown that RapZ itself represents the GlcN6P sensor in this circuit (Khan et al. 2020). When accumulating in a GlcN6P-free state, RapZ binds and stimulates activity of the two-component system QseE/QseF, which in turn activates transcription of *glmY*. Thus, RapZ increases GlmY amounts to sequester itself, counteracting interaction with GlmZ. Overall, this mechanism achieves GlcN6P homeostasis through feedback regulation of GlmS synthesis, ensuring cell envelope integrity (Khan and Görke 2020).

RapZ forms a tetramer composed of a swapped dimer of dimers (Gonzalez et al. 2017). The protomer consists of two globular domains (RapZ_NTD_: residues 1–152 and RapZ_CTD_: residues 153–284) connected by a linker. The tetramer is maintained through homotypic (CTD/CTD, NTD/NTD) as well as heterotypic (NTD/CTD) self-interactions. Their disruption abolishes *glmS* regulation suggesting that tetrameric RapZ and RNase E form an encounter complex to achieve cleavage of the presumably sandwiched sRNA GlmZ. The RNA-binding domain is apparently formed by the 19 carboxy-terminal residues, which are surface exposed and enriched in positively charged amino acids (Göpel et al. 2013; Gonzalez et al. 2017). The RapZ_CTD_ can dimerize and bind RNA on its own.

The mode of operation employed by RapZ remained mysterious. RNase E is unable to cleave GlmZ in the absence of RapZ, reflecting that GlmZ does not fulfill the requirements for either of the two modes of RNase E entry: The 5′ end of GlmZ is inaccessible and its phosphorylation state has no impact on cleavage (Göpel et al. 2016). Moreover, there is only one single-stranded region that could provide a handhold for RNase E direct entry. Therefore, bypassing the need for a second handhold by simultaneous binding of GlmZ and RNase E, or refolding of GlmZ to make the cleavage site accessible were considered as feasible mechanisms underlying RapZ function (Göpel et al. 2016). In the current study, we explored the mechanistic basis of RapZ activity. By making use of RapZ mutants either affected in binding RNA or RNase E, we reveal a novel mode of RNase E regulation, wherein a protein cofactor (RapZ) activates RNase E through interaction to cleave a transcript (GlmZ) that is recruited by the RNA-binding domain.

## RESULTS

### RapZ multimerization is a prerequisite for interaction with RNase E

In order to pinpoint a region in RapZ that binds RNase E, we first assessed the interaction properties of the separated globular domains of RapZ using the bacterial adenylate cyclase two-hybrid system (BACTH). BACTH relies on reconstitution of cAMP synthesis through interaction of candidate proteins fused to the separately encoded T25- and T18-fragments of a split adenylate cyclase (Karimova et al. 1998). β-Galactosidase activity assays indicated interaction of full-length RapZ (RapZ_FL_) with RNase E as observed previously (Göpel et al. 2013), whereas the separated NTD and CTD lost this ability (Fig. 1A, columns 1–5). Similar results were obtained when adenylate cyclase fragments were swapped between fusion partners (Fig. 1A, columns 6–8). Next, we attempted to identify the shortest RapZ variant able to interact with RNase E. Randomly sized *rapZ* DNA fragments were generated by fragmentase treatment and shotgun cloned, yielding a plasmid library encoding T25-RapZ variants of variable length. The library was screened for variants retaining interaction with T18-RNase E, yielding blue colonies on X-Gal plates (Fig. 1B). Analysis of these colonies by PCR recovered only inserts corresponding in length roughly to full-length *rapZ* (Fig. 1B). In contrast, analysis of white colonies also retrieved shorter *rapZ* fragments, confirming their presence in the library (Fig. 1B; Supplemental Fig. S1A). These observations suggest that virtually the complete RapZ sequence is required for interaction and that removal of few residues from either terminus abolishes binding to RNase E.

To confirm this conclusion, we designed RapZ variants lacking 5 or 10 residues from their amino- or carboxy-termini. Among them, only the RapZ variant lacking the 5 carboxy-terminal residues (RapZ_1–279_) retained interaction with RNase E (Fig. 1C, upper panel). To further dissect the interaction requirements, three additional RapZ truncations lacking 6, 7, or 8 residues from the carboxyl terminus were tested. None of these variants was capable of efficiently contacting RNase E (Fig. 1C, upper panel), albeit the RapZ_1–278_ variant retained residual interaction potential, when adenylate cyclase domains were swapped (Supplemental Fig. S1B). We previously showed that RapZ requires oligomerization for activity, that is, substitutions of residues involved in inter-domain interactions concomitantly abrogate regulation of *glmS* (Gonzalez et al. 2017). Indeed, according to BACTH, the amino-terminally truncated variants are unable to self-oligomerize and this also applies to the variants lacking 9 and 10 residues from the carboxyl terminus (Fig. 1C, bottom panel). Hence, loss of interaction with RNase E as observed for these RapZ truncations could be due to their inability to self-interact. Among the carboxy-terminally truncated variants retaining self-interaction, only RapZ_1–279_ was able to bind RNase E, but not RapZ_1–278_ and RapZ_1–277_ (Fig. 1C, compare upper and bottom panel; Supplemental Fig. S1B). To dissect the roles of residues 279 and 278 further, each of them was substituted with a glycine in the context of the RapZ_1–279_ variant. As expected, these substitutions did not affect self-oligomerization confirming that both residues are dispensable for RapZ multimerization (Fig. 1D, bottom panel). However, interaction with RNase E was somewhat decreased upon substitution of Thr278 and abolished in the case of the Leu279Gly substitution (Fig. 1D, top panel). We conclude that Leu279 is critical for and Thr278 may support efficient interaction of RapZ with RNase E.

In an attempt to identify additional residues involved in binding RNase E, we randomly mutagenized *rapZ* using error prone PCR and screened for variants exhibiting impaired interaction in the context of BACTH. Several such mutants carrying single amino acid substitutions were isolated (Supplemental Fig. S2A; Supplemental Table S1). Reporter gene assays addressing the ability to complement an endogenous *rapZ* deletion showed that the RapZ mutants are largely inactive (Supplemental Fig. S2B), which was confirmed by analyzing GlmS protein levels in total protein extracts (Supplemental Fig. S2C). These results corroborate that undisturbed interaction of RapZ with RNase E is essential for regulation of GlmS synthesis. However, the identified substitutions scatter across the RapZ sequence and fail to form a defined surface (Supplemental Fig. S3; Supplemental Table S1). Interestingly, the Val29 and Leu36 residues hit in the screen were previously shown to contribute to the RapZ NTD/NTD self-interaction (Gonzalez et al. 2017). Moreover, the screen-derived substitutions located in the RapZ_CTD_ invariably impaired dimerization of this domain (Supplemental Fig. S2D), collectively corroborating the idea that loss of interaction with RNase E might be an indirect consequence of a disturbed self-assembly of the various RapZ mutants. We conclude that interaction with RNase E requires undisturbed multimerization of RapZ and that residues from its NTD as well as CTD including Leu279 contribute to RNase E binding.

### RapZ interacts with the large amino-terminal globular domain of RNase E

We previously demonstrated interaction of RapZ with an RNase E variant (aa 1–597) comprising the amino-terminal catalytic domain (Göpel et al. 2013). To map the interaction surface in detail, we tested additional RNase E truncations using BACTH. RapZ interacted with full-length RNase E as well as two shorter variants (RNase E_1–597_ and RNase E_1–762_) still comprising the entire catalytic domain (Fig. 1E), as expected. Of the even shorter variants, RNase E_1–415_ and RNase E_1–400_ retained interaction, indicating that the small domain and the Zn-link are dispensable for binding RapZ. In agreement, an RNase E_416–1061_ variant failed to interact confirming that the small domain does not bind RapZ (Supplemental Fig. S4). Removal of subdomains from the large domain abolished interaction (Fig. 1E). Thus, RapZ requires the complete large globular subdomain in the RNase E catalytic domain for interaction.

For further insight, a random mutagenesis screen in the context of BACTH was performed as described for RapZ, but in this case residues 1–497 of the Rne_NTD_ were mutagenized. Altogether, 10 Rne_NTD_ mutants carrying different single amino acid substitutions and exhibiting diminished interaction with RapZ were isolated (Supplemental Fig. S5; Supplemental Table S2). Five of these mutants carried proline substitutions suggesting that structural distortion abrogated interaction. This may also hold true for the G66S and L68F substitutions, which were previously isolated in an independent study and implicated in destabilization of the S1 subdomain, conferring loss of RNase E functionality at high temperature (McDowall et al. 1993; Schubert et al. 2004). Interestingly, we also isolated two mutants which carried an in-frame deletion of residues 170–180 in addition to a R169L substitution (Supplemental Table S2). These residues are part of the 5′-monophosphate sensing pocket and Arg169 directly engages in binding this moiety (Callaghan et al. 2005). Collectively, these data suggest that RapZ contacts multiple sites in the RNase E large globular subdomain, perhaps also including the monophosphate sensing pocket.

### RNA is dispensable for RapZ/RNase E complex formation

**FIGURE 2.**
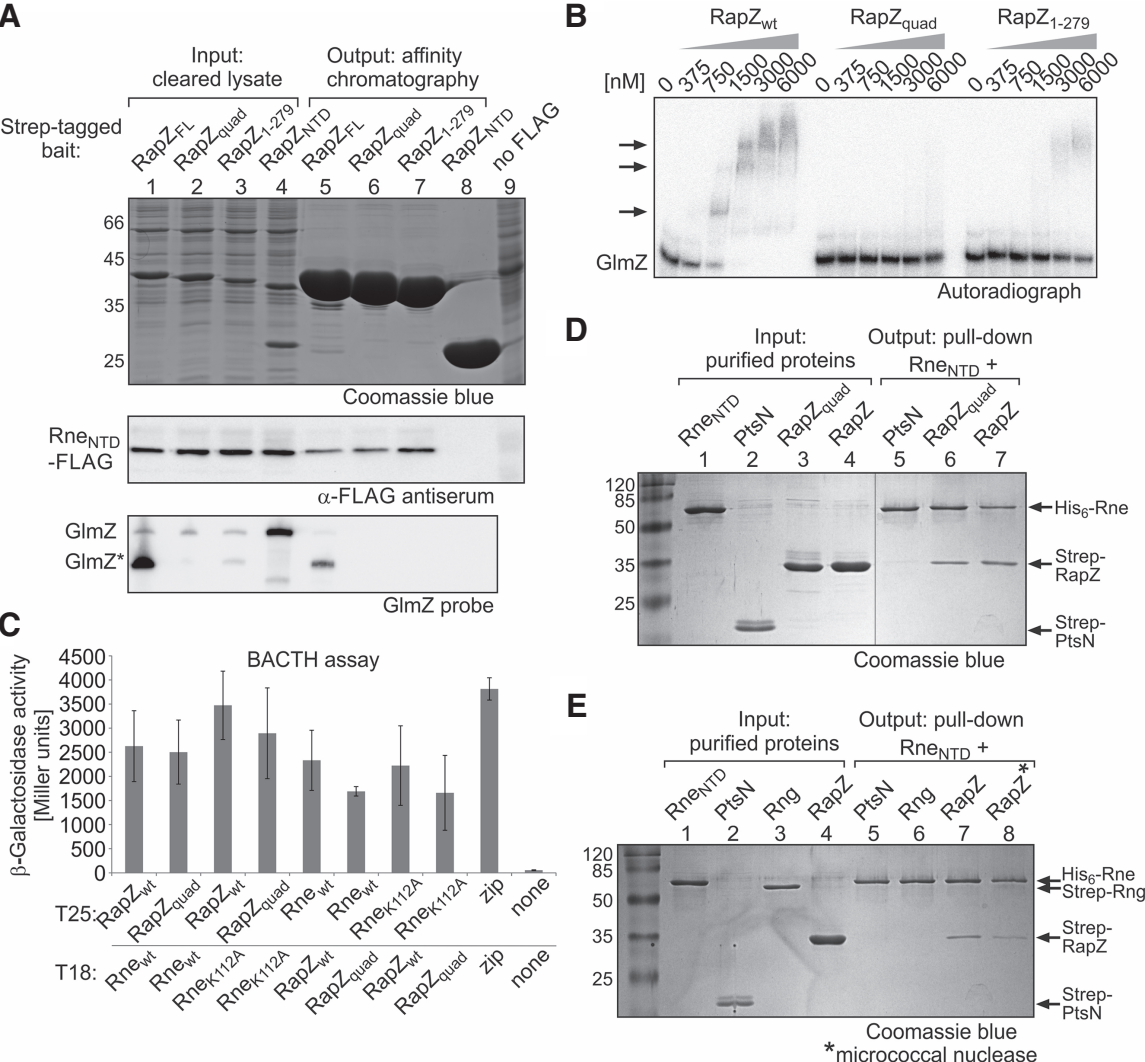
RNA is not required for RapZ/RNase E interaction. (*A*) Ligand fishing experiment based on StrepTactin affinity chromatography indicating that the RNA-binding function of RapZ is dispensable for pull-down of the Rne_NTD_. Strain Z903, which encodes a FLAG-tagged version of Rne_NTD_ in the chromosome and lacks endogenous *rapZ* was transformed with the following plasmids enabling overproduction of the indicated bait protein, respectively: pBGG164 (Strep-RapZ_FL_), pYG29 (Strep-RapZ_quad_), pSD135 (Strep-RapZ_1–279_), and pSD25 (Strep-RapZ_NTD_). Cleared lysates were prepared (lanes *1*–*4*; “input”) and subjected to StrepTactin affinity chromatography. Elution fractions (normalized to protein content; lanes *5*–*8*) were separated on SDS-PAA gels and stained with Coomassie blue to verify successful bait protein purification (*top* panel) and subjected to Western analysis using anti-FLAG antiserum for detection of copurifying Rne_NTD_ (*medium* panel). RNA was extracted from the elution fractions and analyzed by northern blotting for presence of copurified GlmZ (*bottom* panel). Strain Z37 lacking the FLAG epitope served as negative control (last lane). (*B*) EMSA comparing the RNA-binding activities of RapZ, RapZ_quad_, and RapZ_1–279_. α-^32^P-UTP labeled GlmZ was incubated with increasing concentrations of the indicated RapZ variant and reactions were separated on non-denaturing PAA gels and analyzed by phospho-imaging. (*C*) Mutations abolishing the RNA-binding activities of RapZ and Rne_NTD_ do not interfere with interaction as measured by BACTH. Tested plasmids were pBGG348 (T25-RapZ), pYG94 (T25-RapZ_quad_), pYG101 (T25-Rne_NTD_), pYG202 (T25-Rne_NTD_-K112A), pYG97 (T18-Rne_NTD_), pYG201 (T18-Rne_NTD_-K112A), pBGG349 (T18-RapZ), and pYG39 (T18-RapZ_quad_). Plasmid combinations pKT25-zip/pUT18C-zip and pKT25/pUT18C served as positive and negative controls, respectively. β-galactosidase activities are presented as mean ± SD. *n* ≥ 3. Two-tailed Student's *t*-tests were performed to assess whether two data sets are significantly different. The calculated *P*-values are reported in the source data file (Supplemental Table S3). (*D*) In vitro pull-down assays to probe Rne_NTD_/RapZ interaction. Following its immobilization on Ni-NTA magnetic agarose beads, His_6_-Rne_NTD_ was incubated with Strep-PtsN, Strep-RapZ_quad_, or RapZ and bound protein fractions were analyzed by SDS-PAGE/Coomassie blue staining (lanes *5*–*7*). Purified protein preparations (“input”) were analyzed in lanes *1*–*4*. (*E*) Similar approach as in (*D*) but with additional controls including Strep-RNase G as prey. Moreover, His_6_-Rne_NTD_ and Strep-RapZ preparations were treated with micrococcal nuclease prior to their coincubation (last lane). In lane *7*, both proteins were treated similarly but micrococcal nuclease was omitted. Note that except for His_6_-Rne_NTD_ all proteins used in (*D*) and (*E*) were purified from strain Z106 (*ΔglmY ΔglmZ*) to exclude copurification of the sRNAs.

Our mutational analysis of RapZ has shown that residue Leu279 is important for binding RNase E but dispensable for self-interaction (Fig. 1C,D). However, this residue is located in the RNA-binding domain of RapZ (Supplemental Fig. S3), leaving open whether RapZ/RNase E interaction is direct or mediated through bound RNA. For clarification, we tested the ability of selected RapZ variants to bind RNase E and sRNA GlmZ by pull-down experiments using a strain lacking endogenous *rapZ* and carrying a chromosomally encoded RNase E (aa 1–598)-FLAG (Rne_1–598_) variant. A control experiment shows that Rne_1–598_ cleaves GlmZ in a RapZ dependent manner in vivo with virtually no difference to full-length RNase E (Supplemental Fig. S6). Accordingly, the scaffolding domain of RNase E and degradosome formation do not play a role in the GlmY/GlmZ/*glmS* circuit. Among the various truncations, only RapZ_1–279_ preserved interaction with RNase E (Fig. 1C). However, RapZ_1–279_ lacks five residues of the carboxy-terminal RNA-binding domain, potentially affecting RNA-binding. Indeed, pull-down experiments based on StrepTactin affinity chromatography of cell lysates showed that RapZ_1–279_ is able to bind Rne_1–598_, as expected, whereas no GlmZ could be detected in the pull-down fraction (Fig. 2A). We obtained a similar result for a RapZ variant (RapZ_quad_), which carries a quadruple substitution in the RNA-binding domain (K270A, K281A, R282A, K283A) abolishing RNA-binding activity as demonstrated previously (Supplemental Fig. S3; Göpel et al. 2013). In contrast, Rne_1–598_ and GlmZ* were both detectable in the pull-down fraction of wild type RapZ, but not when using the RapZ_NTD_ as bait (Fig. 2A). EMSA using purified proteins and radiolabeled GlmZ confirmed that RapZ_quad_ and RapZ_1–279_ are virtually free of or strongly impaired in RNA-binding activity, respectively (Fig. 2B). For RapZ_1–279_, only a minor fraction of GlmZ was bound at the highest protein concentrations (≥3 µM). Nevertheless, this residual RNA-binding activity is not sufficient to bind GlmZ in vivo as indicated by the copurification experiment (Fig. 2A). These results indicated that RNA-binding activity of RapZ is dispensable for complex formation with RNase E.

We further assessed the potential requirement of RNA for RapZ/RNase E interaction by BACTH. Presence of the quadruple substitution did not affect interaction of RapZ with the Rne_NTD_ (Fig. 2C). To exclude that interaction is mediated by RNA bound to RNase E, we tested an Rne_NTD_ variant carrying the K112A substitution, which abolishes RNA-binding activity and concomitantly reduces the RNA cleavage rate by 98% (Callaghan et al. 2005). However, interaction with RapZ persisted even when both proteins were impaired in their RNA-binding function (Fig. 2C).

Finally, we performed in vitro pull-down assays using purified proteins (Fig. 2D, lanes 1–4). The Strep-tagged candidate proteins were coincubated with the His_6_-tagged Rne_NTD_, which was immobilized on Ni-NTA magnetic agarose beads. Analysis of the bound protein fraction detected both RapZ as well as RapZ_quad_ together with the Rne_NTD_ in the respective eluates, whereas the unrelated protein PtsN was not retained (Fig. 2D, lanes 5–7). To exclude that interaction was mediated by RNA trace amounts bound by Rne_NTD_, additional controls were performed. We included Strep-tagged RNase G, which also binds RNA (Richards and Belasco 2016). However, RNase G was not retained on the beads (Fig. 2E, lanes 5,6). Moreover, we treated Rne_NTD_ and RapZ preparations with micrococcal nuclease to remove all RNA traces prior to the pull-down assay. Nonetheless, RapZ remained bound to the Rne_NTD_ (Fig. 2E, lanes 7,8). We conclude that RapZ/RNase E complex formation involves direct protein–protein interactions and is not merely mediated by RNA.

### The RNA-binding function of RapZ is not sufficient to promote efficient cleavage of GlmZ

Considering the cleavage mechanism, one possibility was that RapZ changes the GlmZ fold, making it amenable for RNase E attack (Göpel et al. 2016). However, our finding that RapZ directly contacts the Rne_NTD_ opened the possibility for an alternative scenario, in which RapZ stimulates RNase E activity through protein–protein interaction, while RNA-binding serves a role in recruiting GlmZ to the complex or orientating the sRNA for proper cleavage.

**FIGURE 3.**
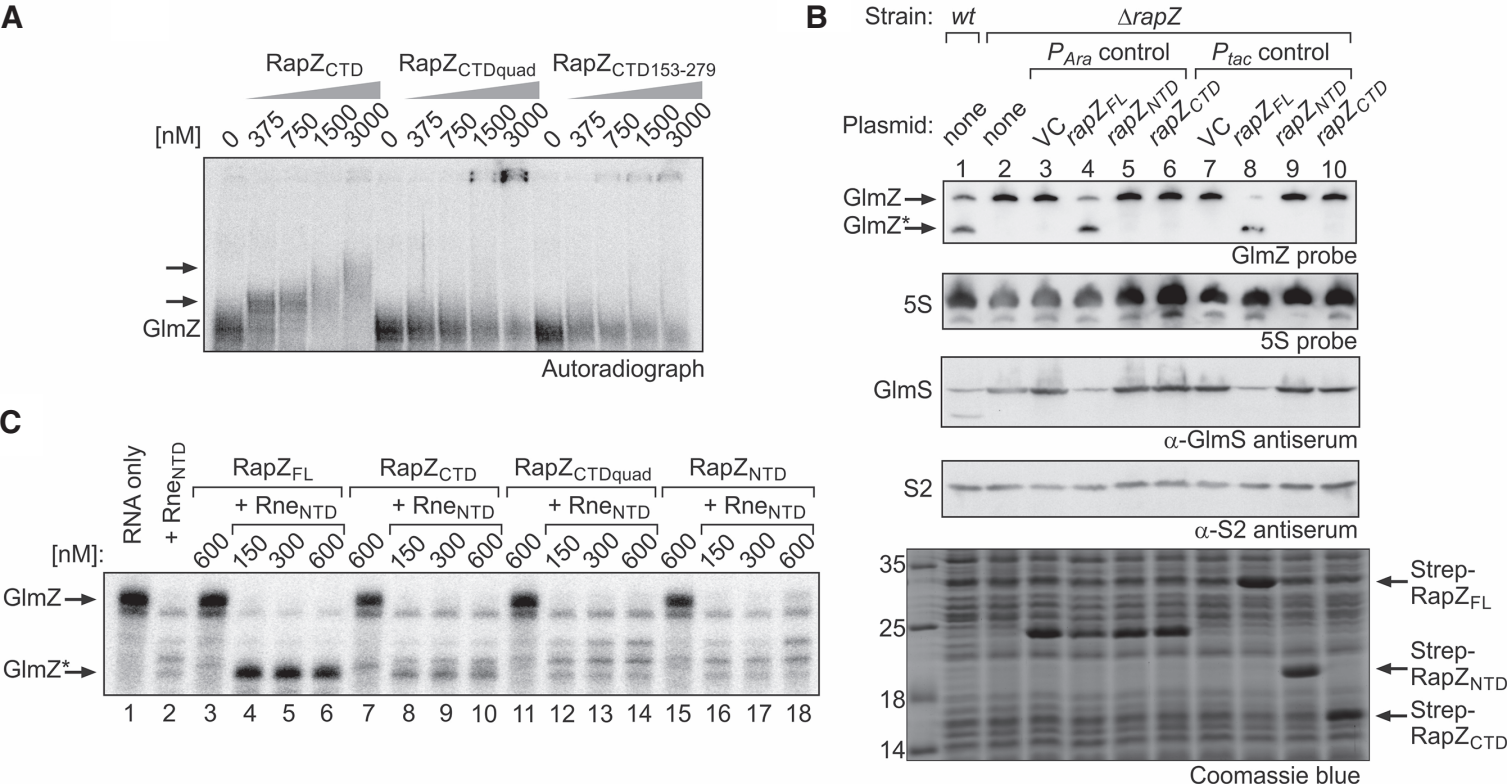
The separated globular domains of RapZ fail to promote GlmZ cleavage in vivo and in vitro. (*A*) EMSA addressing the RNA-binding activity of RapZ_CTD_ and variants thereof carrying a quadruple substitution (K270A, K281A, R282A, K283A) or lacking the 5 carboxy-terminal residues. α-^32^P-UTP labeled GlmZ was incubated with increasing concentrations of the indicated RapZ_CTD_ variants and reactions were analyzed by native PAA gel electrophoresis followed by phospho-imaging. (*B*) Complementation experiment addressing the activities of plasmid-encoded RapZ_NTD_ and RapZ_CTD_ in vivo. In lanes *2*–*10* strain Z37 was used, which lacks endogenous *rapZ*, but carried plasmids triggering either low (lanes *4*–*6*) or high (lanes *8*–*10*) expression levels of encoded *rapZ* variants from the *P*_*Ara*_ or the *P*_*tac*_ promoter, respectively. Low level expression *P*_*Ara*_ constructs were pBAD33 (VC), pBGG61, pSD26, and pSD27. *P*_*tac*_ constructs triggering high expression levels were pBGG237 (VC), pBGG164, pSD25, and pSD24. In the case of the *P*_*Ara*_ constructs, gene expression was induced with 0.2% arabinose. Total RNA and protein were isolated from exponentially growing cells and subjected to northern blotting for detection of GlmZ and 5S rRNA (loading control) and western blotting for detection of GlmS and S2 (loading control) proteins. A Coomassie stained SDS-PAA gel of separated cell extracts is shown at the *bottom* to verify overproduction of the RapZ variants from the *P*_*tac*_ promoter plasmids. The wild type strain R1279 was included for comparison (lane *1*). Note that the band migrating at ∼25 kDa in lanes *3*–*6* likely represents the chloramphenicol acetyltransferase resistance marker (MW = 24.976 kDa) encoded on pBAD33 and its derivatives. (*C*) In vitro RNase E cleavage assays addressing the activities of RapZ_CTD_ and RapZ_NTD_. Radiolabeled GlmZ was coincubated with 50 nM Rne_NTD_ and increasing concentrations of RapZ_FL_ (lanes *4*–*6*), RapZ_CTD_ (lanes *8*–*10*), RapZ_CTDquad_ (lanes *12*–*14*) or the RapZ_NTD_ (lanes *16*–*18*). As negative controls, GlmZ was incubated alone (lane *1*) or with each of the proteins individually (lanes *2*,*3*,*7*,*11*,*15*). Following incubation, reactions were separated on denaturing PAA gels and analyzed by phospho-imaging.

For discrimination, we first assessed the regulatory potential of the RapZ_CTD_, which forms a dimer on its own and retains full RNA-binding activity, but fails to bind RNase E (Figs. 1A, 3A; Gonzalez et al. 2017). Introduction of the quadruple substitution or deletion of the 5 carboxy-terminal residues prevents RapZ_CTD_/GlmZ complex formation as revealed by EMSA using purified proteins and radiolabeled GlmZ (Fig. 3A). This observation suggests that the RapZ_CTD_ dimer binds GlmZ in the same manner employing the same residues as tetrameric full-length RapZ. However, a low copy pBAD33 plasmid expressing *rapZ*_*CTD*_ failed to complement deletion of endogenous *rapZ* (Fig. 3B, lane 6). A similar result was obtained when using a plasmid encoding the RapZ_NTD_, which lacks both RNA-binding activity and RNase E interaction potential (Fig. 3B, lane 5; Gonzalez et al. 2017). In both cases, GlmZ remained unprocessed, triggering accumulation of GlmS to high levels (Fig. 3B, lanes 1–6). We performed a follow-up experiment but used plasmids directing strong overproduction of the RapZ variants to levels becoming visible upon Coomassie staining of gel-separated total protein extracts (Fig. 3B, bottom panel). However, similar results were obtained, indicating that the RapZ_CTD_ is unable to stimulate GlmZ processing in vivo even when present at high levels (Fig. 3B, lanes 7–10).

For confirmation, we made use of a previously established RNase E cleavage assay (Göpel et al. 2013, 2016; Durica-Mitic and Görke 2019) to analyze activity of the RapZ_CTD_ in vitro. In this assay, correct cleavage of radiolabeled GlmZ yielding the 155 nt long 5′ cleavage product (designated GlmZ*) only occurs in the presence of both RapZ and Rne_NTD_ (Fig. 3C, lanes 1–6). Incubation with solely Rne_NTD_ results in accumulation of unspecific cleavage products, whereas RapZ alone has no effect (Fig. 3C, lanes 1–3). When the RapZ_NTD_ was used in the assay, only unspecific cleavage occurred, confirming that this domain cannot guide RNase E (Fig. 3C, lanes 15–18). Interestingly, presence of the RapZ_CTD_ reduced unspecific cleavage by RNase E and led to accumulation of GlmZ* but to a drastically diminished extent when compared to full-length RapZ (Fig. 3C, lanes 3–10). Introduction of the quadruple mutation into RapZ_CTD_ abrogating its RNA-binding activity suppressed residual accumulation of GlmZ* (Fig. 3C, lanes 11–14). Thus, RNA-binding per se can mediate correct cleavage of GlmZ to a minor extent, but does not lead to a boost of RNase E activity as observed for full-length RapZ. Apparently, the RapZ_CTD_ acts in a passive manner only, most likely by occluding unspecific cleavage sites upon binding. These data argue against a mechanism in which RapZ would merely act by making the cleavage site in GlmZ accessible for RNase E. This conclusion is also supported by previous structural probing experiments, which did not yield evidence for GlmZ structure rearrangements upon RapZ binding (Göpel et al. 2013).

### RapZ variants impaired in RNA-binding but retaining interaction with RNase E promote GlmZ processing in vitro

**FIGURE 4.**
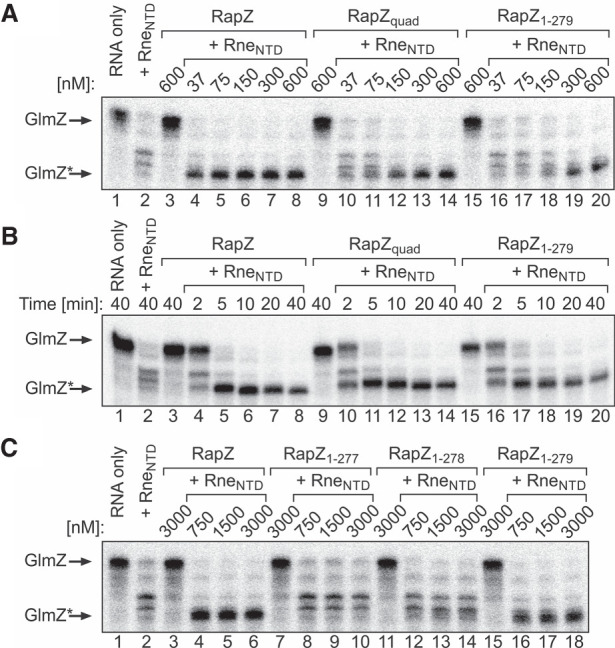
RapZ variants lacking RNA-binding activity but retaining interaction with RNase E promote cleavage of GlmZ by RNase E in vitro. (*A*) In vitro RNase E cleavage end point assays addressing the activities of the RapZ_quad_ and RapZ_1–279_ variants. Radiolabeled GlmZ was coincubated with 50 nM Rne_NTD_ and incremental concentrations of RapZ, RapZ_quad_, or RapZ_1–279_ for 40 min. To provide controls, GlmZ was incubated alone (lane *1*) or with each of the proteins individually (lanes *2*,*3*,*9*,*15*). Reactions were separated on denaturing PAA gels and analyzed by phospho-imaging. (*B*) Time course of GlmZ cleavage by Rne_NTD_ (50 nM) in presence of 300 nM RapZ, RapZ_quad_ or RapZ_1–279_. Aliquots were removed and reactions were stopped at indicated times. (*C*) In vitro RNase E cleavage end point assays comparing the activities of the RapZ_1–277_, RapZ_1–278_, and RapZ_1–279_ variants. Radiolabeled GlmZ and indicated proteins were coincubated for 40 min.

To obtain more insight, we assessed the RapZ_quad_ and RapZ_1–279_ variants, which retain interaction with RNase E but are impaired in RNA-binding—the opposite characteristics to RapZ_CTD_. Interestingly, in vitro both RapZ variants promoted conversion of full-length GlmZ to GlmZ* within the reaction time when present at concentrations ≥300 nM (Fig. 4A). Next, we compared the kinetics of GlmZ cleavage in the presence of 300 nM of the various RapZ variants. Aliquots were removed and reactions were stopped after various incubation times. Interestingly, a similar temporal profile of GlmZ processing could be observed for all three RapZ variants, as GlmZ was completely converted to GlmZ* in all cases following 5 min incubation (Fig. 4B). To confirm that interaction with RNase E is required for RapZ activity, we additionally tested the carboxy-terminally truncated RapZ_1–277_ and RapZ_1–278_ variants, which largely lost the potential to interact with RNase E, while self-interaction was retained (Fig. 1C). Indeed, both variants were inactive, whereas the RapZ_1-279_ variant promoted GlmZ cleavage as observed before (Fig. 4C). We conclude that the RNA-binding function of RapZ is dispensable for rapid and correct processing of GlmZ by RNase E in vitro. In contrast, interaction with RNase E is crucial for this activity.

### High protein levels compensate for loss of the RNA-binding function of RapZ in vivo

**FIGURE 5.**
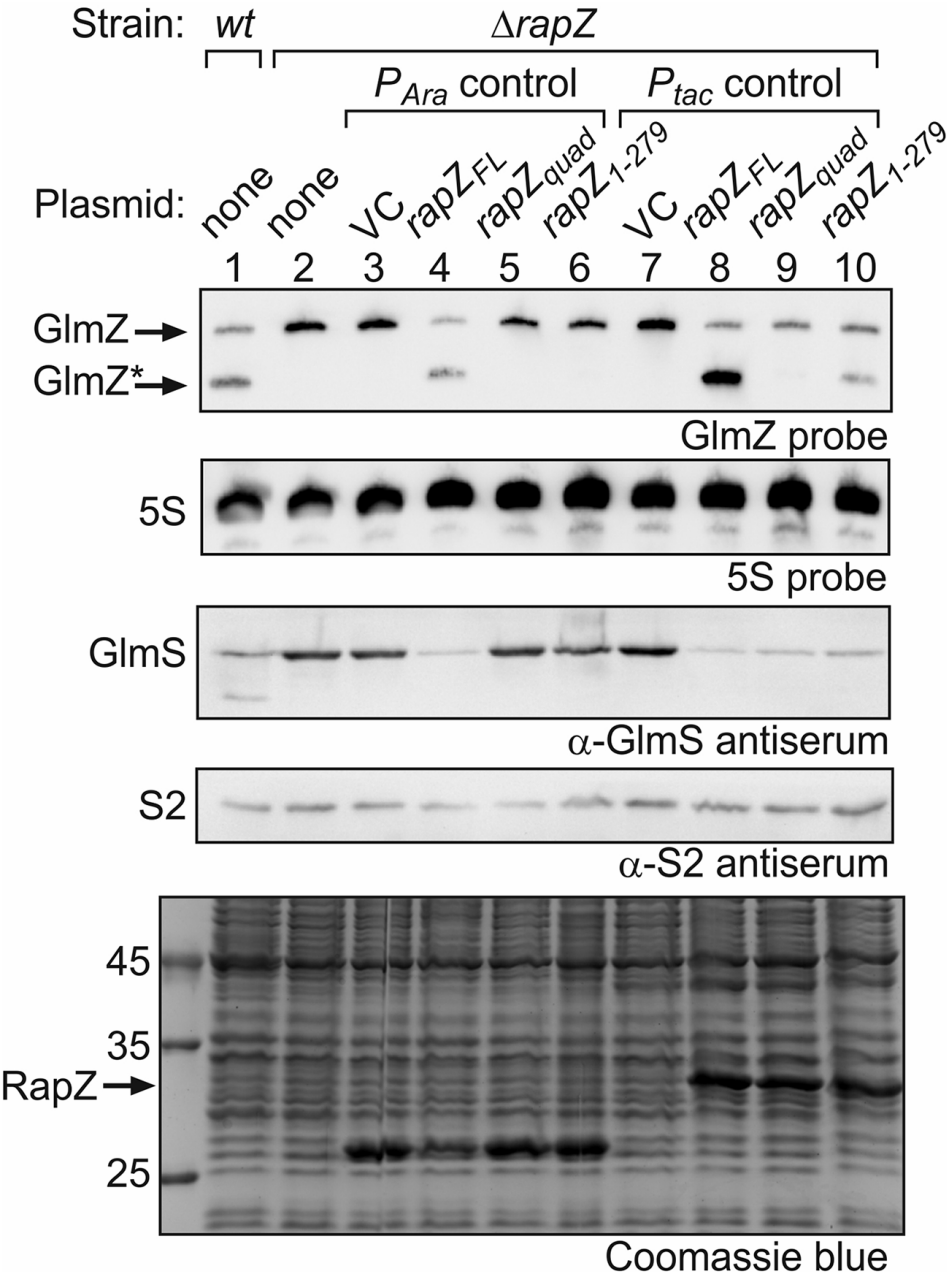
RapZ overproduction may compensate for loss of its RNA-binding activity in vivo. Complementation experiment addressing the activities of plasmid encoded RapZ_quad_ and RapZ_1–279_ variants in vivo. Plasmids triggering either low (lanes *4*–*6*: pBGG61, pYG30, pSD128) or high (lanes *8*–*10*: pBGG164, pYG29, pSD135) expression levels of the *rapZ* variants from the *P*_*Ara*_ or the *P*_*tac*_ promoter, respectively, were tested in strain Z37, which lacks endogenous *rapZ*. Empty plasmids pBAD33 and pBGG237 provided the vector controls, respectively (VC; lanes *3* and *7*). In lane *1*, wild type strain R1279 was analyzed for comparison. For induction of the *P*_*Ara*_ promoter, 0.2% arabinose was added. Total RNA and protein were isolated from exponentially growing cells and analyzed by northern and western blotting, respectively, for detection of GlmZ, 5S rRNA (loading control), GlmS and protein S2 (loading control). In the *bottom* panel, total protein extracts were separated on a SDS-PAA gel and stained with Coomassie blue to verify overproduction of the RapZ variants from the *P*_*tac*_ constructs (indicated by arrows).

Even though RNA-binding activity is not required for RapZ to promote GlmZ processing in vitro (Fig. 4), the contribution of this function may differ in vivo, where a plethora of RNAs compete to gain access to RNase E. To assess this, we tested whether RapZ_quad_ and RapZ_1–279_ are able to complement a strain lacking endogenous *rapZ*. First, we expressed the *rapZ* variants from low copy pBAD33 derivatives, which trigger RapZ levels close to physiological levels upon induction with arabinose (Göpel et al. 2013; Durica-Mitic and Görke 2019). Complementation with the plasmid producing wild type RapZ perfectly restored GlmZ processing, thus suppressing GlmS synthesis (Fig. 5, lanes 1–4). However, in the presence of the plasmids encoding RapZ_quad_ or RapZ_1–279_, GlmZ remained unprocessed and accumulated, thereby up-regulating GlmS to levels observed in the non-complemented strain (Fig. 5, lanes 5,6).

Higher concentrations of RapZ_quad_ and RapZ_1–279_ compared to wild type RapZ are required in vitro to achieve GlmZ processing (Fig. 4A). To mimic these conditions in vivo, we used vectors directing the strong overproduction of the individual RapZ variants (Fig. 5, lanes 8–10, bottom panel). Indeed, under these conditions, the presence of RapZ_quad_ or RapZ_1–279_ reduced the levels of full-length GlmZ and concomitantly abolished accumulation of GlmS. However, processed GlmZ* accumulated to lower levels or was almost undetectable when compared to the strain overproducing wild type RapZ (Fig. 5, lanes 7–10). We recently demonstrated that GlmZ* likewise binds RapZ, thereby preventing complete turnover of full-length GlmZ, assuring a robust basal level of *glmS* expression (Durica-Mitic and Görke 2019). Likely, binding by RapZ protects GlmZ* from further degradation, explaining the decreased GlmZ* levels observed in the case of the RapZ_quad_ or RapZ_1–279_ variants. Taken together, we conclude that the RNA-binding function of RapZ is essential for cleavage of GlmZ when RapZ is produced at physiological levels. However, similar to the observations in vitro (Fig. 4A), loss of RNA-binding can be compensated by elevating RapZ amounts.

### Impact of RapZ on cleavage of non-cognate transcripts by RNase E in vitro

**FIGURE 6.**
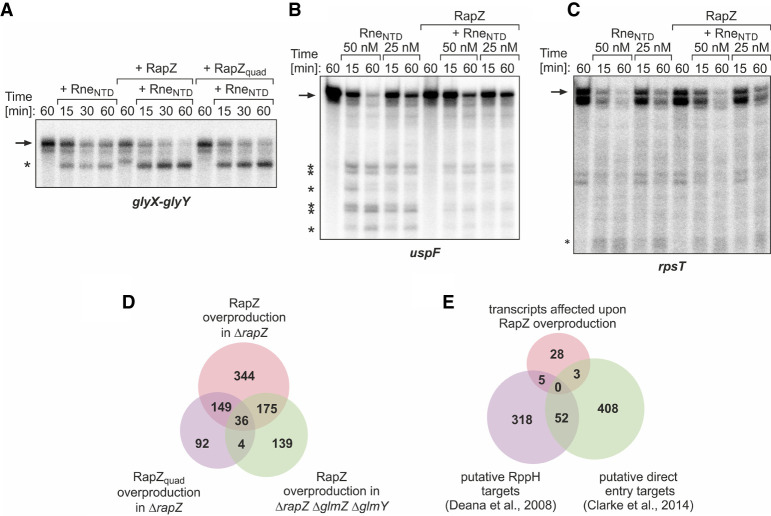
Impact of RapZ on cleavage of non-cognate transcripts by RNase E. Time course analysis of cleavage of *glyX–glyY* (*A*), *uspF* (*B*), and *rpsT* (*C*) by Rne_NTD_ in absence and presence of RapZ in vitro. Where indicated, 600 nM RapZ or RapZ_quad_ was added to the assay. Rne_NTD_ concentrations were 5 nM for *glyX–glyY* cleavage assays and 50 or 25 nM for assays addressing turnover of *uspF* and *rpsT* transcripts. Samples were removed and reactions were stopped at indicated times. Reactions were separated by denaturing gel electrophoresis and analyzed by phospho-imaging. Cleavage products are indicated with asterisks. (*D*) Venn diagram illustrating the result of the RNA-seq analyses addressing the effect of RapZ overproduction on the transcriptome. Shown are the number of transcripts affected by overproduction of RapZ and RapZ_quad_ in strain Z37 (*ΔrapZ*) and of RapZ in strain Z864 (*ΔrapZ ΔglmY ΔglmZ*) as compared to the untransformed strains. A total of 36 transcript changes are shared by the three conditions and therefore considered to be directly caused by RapZ independent of its RNA-binding function (see also Supplemental Table S8). (*E*) The 36 transcripts in the intersection from (*D*) were compared with previous data sets reporting putative RppH targets (Deana et al. 2008) and potential direct entry RNase E substrates (Clarke et al. 2014).

Our data indicated that RapZ stimulates GlmZ processing by a mechanism that relies on direct interaction with RNase E. This observation raised the question whether RapZ could also stimulate cleavage of RNase E substrates beyond GlmZ. Hence, we tested decay of several transcripts by RNase E in vitro, including the direct entry substrates *glyX–glyY* and *uspF* (Clarke et al. 2014) as well as *rpsT*, whose degradation can be initiated via both pathways (Luciano et al. 2012; Mackie 2013a). The *glyX–glyY* transcript was not completely cleaved within 60 min when incubated with 5 nM Rne_NTD_ (Fig. 6A). However, in the additional presence of RapZ, cleavage was accelerated and a similar result was obtained when using the RapZ_quad_ variant, indicating that RNA-binding activity is dispensable for this stimulatory effect (Fig. 6A). In contrast, RapZ had no role for degradation of the *rpsT* RNA by RNase E (note that in vitro transcription generated two *rpsT* mRNA variants), and in the case of the *uspF* mRNA it even appeared to act inhibitory on degradation (Fig. 6B,C). Thus, RapZ can stimulate RNase E to cleave *glyX–glyY* RNA, at least when provided at high concentrations in vitro. However, this does not apply to every RNA substrate, indicating that certain features within the transcript are also important, allowing RapZ to act.

### Stimulation of RNase E activity by endogenously encoded RapZ is restricted to GlmZ and does not affect other RNase E substrates in vivo

Our results have shown that RNA-binding activity is not strictly required for RapZ to stimulate cleavage of GlmZ and the non-cognate *glyX–glyY* RNA in vitro (Figs. 4A,B, 6A). This opens the possibility that RapZ stimulates RNase E activity through protein–protein interaction to cleave additional RNAs in vivo. To address this, we studied the impact of a *rapZ* deletion on the whole transcriptome by RNA-seq. To account for transcripts that are indirectly affected by the *ΔrapZ* mutation through the sRNAs, *ΔglmY ΔglmZ* double and *ΔrapZ ΔglmY ΔglmZ* triple mutants were analyzed as well. When focusing on transcripts changed at least twofold in abundance (|log_2_ fold change| ≥ 1.0; adjusted *P*-value ≤ 0.05), 29 genes were identified whose expression was altered in the *ΔrapZ* mutant (Table 1; Supplemental Table S4). The *rapZ* transcript was strongly down-regulated confirming its absence in this strain. Accordingly, GlmZ was ninefold up-regulated reflecting block of its degradation and concomitantly *glmS* was strongly up-regulated as expected. Northern blot analysis confirmed selective up-regulation of *glmS* in the *ΔrapZ* mutant (Supplemental Fig. S7A). Across strains, 15 additional genes were exclusively affected in the *ΔrapZ* mutant suggesting that they are regulated by RapZ through the sRNA(s) similar to *glmS*. In addition to *ybhH, opgB*, *trxB, hcaB*, and *lysU*, this group included several transcripts related to iron uptake (*entCEBH*, *fepA, fhuB, cirA, fiu, fhuCD*). However, *entC* and *cirA* mRNAs, which were selected for validation by northern blotting, were only detectable under iron starvation conditions and unaffected by RapZ (Supplemental Fig. S7B,C). Hence, only basal expression levels of iron acquisition genes might be affected, perhaps through pleiotropic effects generated by chronic overproduction of GlmS in the *ΔrapZ* mutant. Additionally, transcripts for type 1 fimbriae (pili) synthesis (*fimAICDFG*) were up-regulated in the *ΔrapZ* mutant and surprisingly even stronger in the *ΔglmY ΔglmZ* double mutant (Table 1; Supplemental Table S5). However, the *fimA* promoter is known to undergo spontaneous site-specific inversion (Abraham et al. 1985). Indeed, PCR analysis of the RNA samples subjected to RNA-seq (collected prior to the DNA removal step) revealed a switch of the *fimA* promoter from the “OFF” state as observed in the wild type ancestor strain into the “ON” configuration. This switch occurred either in a minority (*ΔrapZ* strain) or majority (*ΔglmY ΔglmZ* strain) of cells, correlating with the mean read numbers obtained by RNA-seq (Supplemental Fig. S8). Consequently, up-regulation of the *fimAICDFG* operon cannot be attributed to RapZ or the sRNAs. Finally, *yeeR* and *flu* were the only transcripts, which changed in the *ΔrapZ* and the *ΔrapZ ΔglmY ΔglmZ* mutants, but remained unaffected in the *ΔglmY ΔglmZ* strain, as expected for transcripts directly regulated by RapZ not involving the sRNAs (Tables 1, 2; Supplemental Tables S4, S5). This result shows that RapZ does not have a prominent role in turnover of transcripts that are degraded by RNase E beyond GlmZ, at least when produced at physiological levels.

**TABLE 1.**
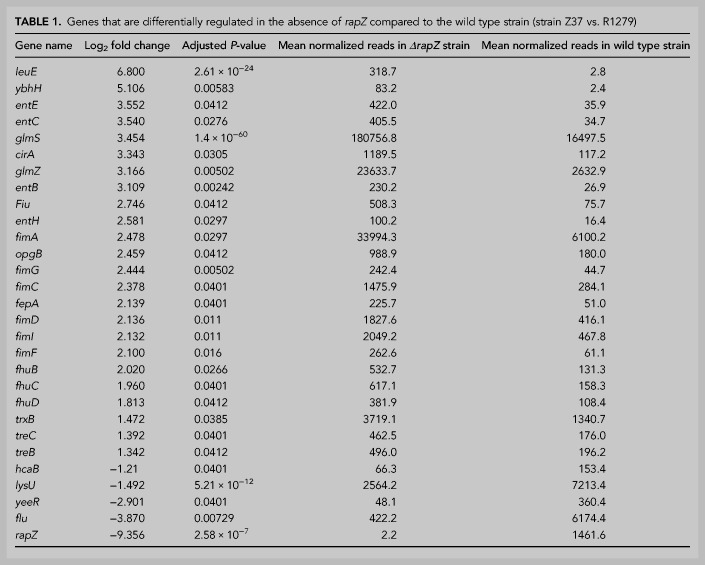
Genes that are differentially regulated in the absence of *rapZ* compared to the wild type strain (strain Z37 vs. R1279)

We made additional observations worth mentioning in the RNA-seq analyses. A few transcripts changed in all mutant strains including *leuE* and maltose (*mal*) utilization genes (note that *mal* genes are not included in Table 1 and Supplemental Table S5 as adjusted *P*-values are >0.05, cf. Supplemental Table S4), but northern blot analysis of *malQ* could not confirm such a change (Supplemental Fig. S7D). Moreover, a couple of cold-shock-related transcripts (*cspI, cspH, cspB, cspF, cspG*, *ydfK*, *ynaE*) were up-regulated in the *ΔglmY ΔglmZ* strain and flagellar-related transcripts (*flgB*, *flgD*, *flgF*, *fliF*, *fliL*, *fliA*) were down-regulated in both the double and triple mutant strain (Table 2; Supplemental Table S5; note that *flgD*, *flgF*, *fliF* and *fliL* are not included in Table 2 as adjusted *P*-values are >0.05). Northern blot analysis could not detect the *fliA* transcript (data not shown), but confirmed accumulation of *cspB* and *cspH* mRNAs in the *ΔglmY ΔglmZ* mutant (Supplemental Fig. S7E,F). Thus, it is possible that GlmY/GlmZ play a role in motility as previously suggested (Bak et al. 2015), and/or the cold shock response.

**TABLE 2.**
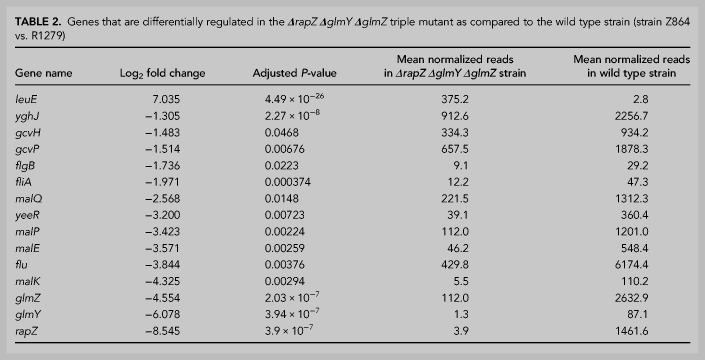
Genes that are differentially regulated in the ***Δ****rapZ* ***Δ****glmY* ***Δ****glmZ* triple mutant as compared to the wild type strain (strain Z864 vs. R1279)

### RapZ is unable to stimulate RNase E activity globally when overproduced

Next, we tested by RNA-seq whether RapZ affects RNase E activity through protein–protein interaction when overproduced. To this end, we used the transformants from above (Fig. 5) triggering the strong overproduction of RapZ and RapZ_quad_, and also included a strain overproducing RapZ in the *ΔrapZ ΔglmY ΔglmZ* mutant to account for indirect changes exerted through GlmY and GlmZ. To assess transcriptome changes elicited by the backbone of the plasmids overexpressing the *rapZ* variants, we determined the effect of the corresponding empty plasmid pBGG237 on the transcriptomes compared to the untransformed strains. According to RNA-seq, presence of the empty plasmid changed expression of 1395 and 1278 genes (|log_2_ fold change| ≥ 1.0; adjusted *P*-value ≤ 0.05) in the two mutants, respectively, exhibiting an overlap of 1021 genes (Supplemental Table S6). A severe impact brought about by plasmid constructs, regardless of their genetic content, on host cell expression has also been noted previously (Ceroni et al. 2018). Accordingly, we removed these genes from the data sets, leaving 2797 and 2914 candidate genes in the *ΔrapZ* and *ΔrapZ ΔglmY ΔglmZ* mutants for analysis, respectively. In the remaining list, we identified 704 genes whose expression changed at least greater than or equal to twofold upon RapZ overproduction when compared to the untransformed strain (Supplemental Table S6). Interestingly, *glmS* and GlmZ levels were down-regulated regardless whether RapZ or RapZ_quad_ was overproduced (Supplemental Table S6), confirming our observation that strong overproduction may compensate for loss of RNA-binding activity of RapZ_quad_ (Fig. 5).

Altogether, we retrieved 939 transcripts that changed greater than or equal to twofold in at least one of the three overproducing strains and were not affected by the empty vector (Fig. 6D). Among them, 36 transcripts were regulated in all three strains suggesting that they are regulated by RapZ, independent of its RNA-binding activity and not involving GlmY or GlmZ (Fig. 6D; Supplemental Tables S6, S8). To determine whether the 36 transcripts are enriched in RNase E substrates, we compared them with two data sets reporting RNase E target transcripts in *E. coli*-K12. The first data set comprises transcripts up-regulated in the absence of RNA pyrophosphohydrolase RppH activity, that is, RNAs likely cleaved by RNase E via the 5′-monophosphate dependent pathway (Deana et al. 2008). The second data set contains putative direct entry targets that can be cleaved by an Rne_NTD_ variant impaired in 5′-monophosphate sensing (Clarke et al. 2014; Supplemental Table S7). However, there was no prominent overlap between the two latter data sets and the 36 candidate transcripts regulated by RapZ (Fig. 6E; Supplemental Table S8). Taken together, RapZ is not able to stimulate RNase E activity globally through protein–protein interaction, even when overproduced. These results show that stimulation of RNase E activity by RapZ affects only GlmZ but virtually no other RNase E substrate in the cell.

## DISCUSSION

In this work, we reveal a novel mode for regulation of RNase E. We demonstrate that activity of the RNase E catalytic domain can be boosted by direct interaction with an accessory protein, RapZ, bypassing the need of an RNA 5′ monophosphate group for allosteric activation of the cleavage reaction. This conclusion is drawn from the characteristics of RapZ variants that are still able to stimulate RNase E activity, albeit their RNA-binding activity is impaired. Nonetheless, RapZ is highly specific for GlmZ in vivo, as revealed by RNA-seq, suggesting that transcripts must possess certain properties to be cleaved by the RapZ/RNase E complex.

Mutational analysis indicates that tetramerization of RapZ is a prerequisite for interaction with RNase E, which explains previous observations that correct RapZ oligomerization is essential for regulation of *glmS* (Gonzalez et al. 2017). The mutagenesis screen primarily retrieved substitutions that affected RapZ self-interaction as shown here (Supplemental Fig. S2D) or identified residues (Val29, Leu36) previously shown to contribute to RapZ inter-domain contacts (Gonzalez et al. 2017). Moreover, removal of a few residues from either terminus abolishes RapZ self-interaction and concomitantly interaction with RNase E (Fig. 1B,C). The only variants that retained self-interaction but were impaired in binding RNase E were RapZ_1–278_ and RapZ_1–277_ (Fig. 1C). Mutational analysis revealed that Leu279 is specifically required for interaction with RNase E (Fig. 1D). Although Leu279 is located in the RNA-binding domain, it may directly contact Rne_NTD_ reflecting that interaction is not mediated through RNA (Fig. 2). However, as the RapZ_CTD_ can dimerize properly on its own but is incapable of binding RNase E (Fig. 1A; Gonzalez et al. 2017), residues from the RapZ_NTD_ may likewise be involved in interaction. Previous size exclusion chromatography analysis also failed to detect complex formation between the RapZ_CTD_ and RNase E in vitro (Gonzalez et al. 2017). Thus, RapZ in its tetrameric form interacts directly via multiple residues with the large globular domain of RNase E (Fig. 1E). The latter domain may form dimers on its own, but is unable to tetramerize, which requires the small domain (Ali and Gowrishankar 2020). According to BACTH analysis, the small domain of RNase E is dispensable for interaction with RapZ (Fig. 1E), indicating that a RapZ tetramer may bind a dimer of the Rne_NTD_. This observation suggests an 8:4 stoichiometry of the RapZ:RNase E complex but other scenarios are possible.

In light of the currently known mechanisms employed by RNase E, one possibility is that RapZ mimics a 5′ terminal RNA monophosphate group and directly interacts for example, through a negatively charged residue with the sensor pocket in the large domain of Rne_NTD_. This allosteric interaction may force the Rne_NTD_ into a closed conformation thereby boosting catalytic activity. The identification of the Rne_NTD_ mutant, which lacks 11 residues of the 5′ monophosphate sensor domain and thus cannot bind RapZ (Supplemental Table S2), supports a direct interaction of RapZ with the latter domain. However, substitution of key residues Arg169 or Thr170 employed by RNase E for 5′ monophosphate sensing does not compromise RapZ-stimulated cleavage of GlmZ in vitro or interaction with RapZ as measured by BACTH (Supplemental Fig. S9). This observation rules out that RapZ acts by making direct contact to one of these residues. Alternatively, RapZ could stimulate RNase E activity by interaction with an autoinhibitory pocket that is formed by four acidic residues in the large domain (Bandyra et al. 2018). This autoinhibition motif intrinsically suppresses RNase E cleavage activity and RapZ could counteract this process to boost GlmZ cleavage. Finally, RapZ could act on a GlmZ/RNase E complex as recently described for sRNA RprA, which binds through a duplex region to the Rne_NTD_ (Bandyra et al. 2018). Previous work has shown that RNase E can bind GlmZ on its own and that a tripartite complex forms when RapZ is also present (Gonzalez et al. 2017). Perhaps, GlmZ is not properly oriented when in complex with RNase E alone and interaction with RapZ confers a conformational change allowing the RNase E catalytic center to reach the scissile phophodiester bond.

The lack of RapZ RNA-binding activity can be compensated by elevating concentrations of the respective RapZ variants (Figs. 4A, 5). This observation indicates that RNA-binding has a recruiting role, increasing the local concentration of GlmZ and thereby the likelihood that productive RapZ/GlmZ/RNase E encounter complexes form. Inside the cell, RNase E is attached to the cytoplasmic membrane (Strahl et al. 2015), while GlmZ preferentially resides in the nucleoid and the cytoplasm (Sheng et al. 2017). This differential compartmentalization may emphasize a recruiting role of RapZ, binding GlmZ in the cytoplasm and shuttling it to the membrane for degradation by RNase E. RNA-binding may also enable RapZ to efficiently compete with Hfq for gaining access to GlmZ. Binding of Hfq and cleavage by RapZ/RNase E is mutually exclusive, targeting the sRNA either to base-pairing or decay, respectively (Göpel et al. 2016). Hfq binds GlmZ within the single-stranded region comprising the base-pairing site and thereby blocks access to the overlapping cleavage site. We observed that the RapZ_CTD_, which is unable to bind RNase E but retains RNA-binding activity, still permits correct processing of GlmZ to a very minor extent in vitro (Fig. 3C). The RapZ_CTD_ likely protects GlmZ from cleavage at non-canonical sites, leaving only the genuine cleavage site accessible for RNase E. Nonetheless, this “passive” activity is not sufficient to guide GlmZ processing in vivo, regardless of the *rapZ*_*CTD*_ expression level (Fig. 3B).

Although RapZ is able to boost RNase E activity through interaction, its genuine function is restricted to GlmZ. Among a few RNase E target RNAs tested in vitro, only decay of the direct entry substrate *glyX–glyY* was accelerated by RapZ in vitro (Fig. 6A). In vivo, merely 28 genes were affected by a *rapZ* mutation. However, northern blot analysis could not confirm a role of RapZ for abundance of transcripts related to iron metabolism (Supplemental Fig. S7), and the changes observed for the pili-related *fimAICDFG* transcript must be ascribed to spontaneous inversion of the *fim* promoter (Supplemental Fig. S8). Among the 36 transcripts collectively affected in the various strains overproducing RapZ, only seven were previously annotated as RNase E substrates and also down-regulated as expected for enhanced turnover (Fig. 6D,E; Supplemental Table S8). Thus, even though a tripartite encounter complex may form, certain features within the transcript are also required for cleavage. This is supported by previous findings that sRNA GlmY, albeit perfectly bound by RapZ, is not cleaved by RNase E (Göpel et al. 2016). However, when the central stem–loop is replaced by the corresponding structure from GlmZ, cleavage occurs. In fact, RapZ triggers cleavage of any RNA sequence fused to the 3′ end of the central stem–loop of GlmZ (Göpel et al. 2016). Obviously, only substrates containing the latter structure can be sterically orientated in the encounter complex allowing the RNase E catalytic center to reach the adjacent cleavage site. Presumably, the extensively folded *glyX–glyY* transcript contains a region resembling the GlmZ central stem–loop allowing its cleavage by the encounter complex.

The list of mRNAs collectively changing upon RapZ or RapZ_quad_ overproduction (Fig. 6D; Supplemental Table S8) contains many functions related to the cell envelope, some of which are likely transcribed from promoters controlled by RpoE, the sigma factor governing the cell envelope stress response (i.e., *ugpQ*, *cysZ*, *ybeQ*, *ybgD*, and *ompA*/*ompC* through sRNAs MicA and RybB [Keseler et al. 2017]). How can RapZ affect these transcripts as they are apparently not regulated via RNase E or GlmY/GlmZ? We recently showed that RapZ binds and stimulates activity of the two-component system QseE/QseF from inside the cell to increase expression of its decoy sRNA GlmY when the GlcN6P concentration drops (Khan and Görke 2020; Khan et al. 2020). QseE/QseF in turn control transcription of *glmY* and *rpoE,* which are the only known target genes in *E. coli* K-12 (Klein et al. 2016; Göpel and Görke 2018). Hence, it is possible that RapZ overproduction affects transcript levels more globally through stimulation of QseE/QseF activity, which remains to be addressed. Stimulation of a regulatory two-component system might even represent the evolutionary primordial function of the widely conserved RapZ protein, reflecting that its RNA-binding domain and concurrently also GlmY/GlmZ are restricted to *Enterobacteriaceae* (Supplemental Fig. S3; Göpel et al. 2011, 2013).

Another example for a protein with a comparable function to RapZ is CsrD, which is specifically required for the turnover of sRNAs CsrB/CsrC by RNase E and has only limited roles beyond (Vakulskas et al. 2016; Potts et al. 2018). Similar to RapZ, CsrD-mediated decay only requires the Rne_NTD_, but whether CsrD also binds this domain is unknown. Interestingly, CsrD binds RNA only nonspecifically, emphasizing that specific RNA-binding activity is dispensable for this “adaptor” function as observed for RapZ in the current study. The phage T4 protein Srd accelerates host mRNA degradation by stimulating cleavage activity of RNase E (Qi et al. 2015). Srd was suggested to associate with the Rne_NTD_, raising the possibility that it may activate RNase E by protein–protein interaction similar to RapZ. In conclusion, the Rne_NTD_ might be a hub for regulatory proteins that hijack this general endoribonuclease to re-program its activity. More of such RNase adaptor proteins are likely to be discovered in the future.

## MATERIALS AND METHODS

### Growth conditions, strains, and plasmids

Bacteria were routinely grown in LB medium at 37°C. When needed, antibiotics were added at the following concentrations: ampicillin 100 µg/mL, kanamycin 30 µg/mL, chloramphenicol 15 µg/mL, and spectinomycin at 50 µg/mL. Expression of genes under control of *P*_*Ara*_ was induced with 0.2% arabinose and repressed by 0.1% glucose. Iron depletion was elicited by growing cells in the presence of 200 µM 2,2′-bipyridyl (DIP) and expression of *mal* genes was induced by addition of 0.2% maltose. Established alleles tagged with a resistance marker were moved between strains by general transduction using phage T4GT7 (Wilson et al. 1979). Lists of strains, plasmids and oligonucleotides used in this study are provided in Supplemental Tables S9–S11, respectively. Construction of plasmids is described under Supplemental Material.

### Bacterial adenylate cyclase two-hybrid (BACTH) analysis

The bacterial two-hybrid (BACTH) system allows monitoring of protein–protein interactions in vivo (Karimova et al. 1998). Candidate proteins are fused to the separately encoded T18- and T25-fragments of a split adenylate cyclase. Plasmid constructs encoding the desired T18- and T25-fusion proteins are introduced into strain BTH101, which lacks endogenous *cyaA*. Interaction of candidate proteins results in reconstitution of cAMP synthesis, which is monitored by measuring LacZ activity. For phenotypic assays, bacteria were grown at 28°C on LB-agar plates containing 1 mM IPTG and 40 µg/mL X-gal. Interactions were quantified by measuring β-galactosidase activities from cells grown to stationary phase at 28°C. BTH101 cells producing the unfused T18- and T25-domains (encoded on plasmids pUT18C and pKT25, respectively), served as negative control. The positive control was provided by cells carrying plasmids pUT18C-zip and pKT25-zip, which encode the leucine zipper of yeast protein Gcn4 fused to T18 and T25, respectively.

### β-Galactosidase activity assays

β-Galactosidase activities were determined as described (Miller 1972). Unless otherwise indicated, presented values derive from a minimum of three measurements using at least two independent transformations.

### Generation of a library of *rapZ* truncations for BACTH screening

An amount of 500 ng of a *rapZ* DNA fragment obtained by PCR using primers BG168/BG639, was treated with NEBNext dsDNA Fragmentase (NEB) for 10, 15, and 20 min at 37°C according to manufacturer's instructions and reactions were stopped by addition of EDTA to a final concentration of 0.1 mM. Obtained DNA fragments were blunted using the Fast DNA End Repair Kit (Thermo Fisher Scientific) and subsequently ligated with SmaI-digested plasmid pKT25, resulting in three libraries containing arbitrarily truncated *rapZ* variants fused to the *T25* sequence. These libraries were subsequently used to transform strain BTH101 carrying plasmid pYG99 encoding T18-Rne_FL_. Recombinants were screened on X-gal plates and the *rapZ* inserts of candidate colonies were PCR-amplified using primers BG646/BG647.

### Random mutagenesis of *rapZ* and *rne* and screen for loss of interaction by BACTH

Genes *rapZ* and *rne* were randomly mutagenized by error prone PCR (Zhou et al. 1991) using primers BG646/BG647 (annealing to the pKT25 plasmid backbone) and plasmids pBGG348 and pYG101 as templates, respectively. The mutagenized *rapZ* DNA fragments were inserted between the XbaI/KpnI sites of plasmid pKT25. The mutagenized *rne* fragments were digested with XbaI and AflII and used to replace the XbaI-AflII fragment comprising aa 1–497 of Rne_NTD_ in BACTH plasmid pYG101. The mutant plasmid libraries were used to transform strain BTH101 carrying plasmid pYG99 encoding T18-Rne_FL_ (for screening of *rapZ* mutants) or plasmid pBGG349 encoding T18-RapZ (for screening of Rne_NTD_ mutants), respectively. Plasmids were extracted from colonies exhibiting less intense blue coloration on X-Gal plates and used to transform BTH101 once again to ensure persistence and uniformity of the phenotype. Plasmids passing this test were sequenced and subjected to quantitative BACTH measurements.

### Ligand fishing using StrepTactin affinity chromatography

Copurification experiments addressing interaction of Strep-tagged RapZ variants with GlmZ and FLAG-tagged Rne_NTD_ were performed as described previously (Göpel et al. 2013). Briefly, Strep-tagged bait proteins were overproduced in strain Z903 lacking endogenous *rapZ* and harboring the *rne_NTD_-FLAG* allele on the chromosome. Cells were grown and harvested as described under “protein purification” followed by lysis using the one shot cell disruptor (Constant Systems Ltd.). Eluates derived from StrepTactin affinity chromatography were analyzed by SDS-PAGE/Coomassie blue staining for visualization of the prey proteins and by western blotting for detection of copurifying Rne_NTD_-FLAG. Half of the eluates were used for RNA extraction as described previously and samples of the isolated RNAs (normalized to protein content of the elution fraction) were subjected to northern blotting for detection of GlmZ.

### SDS-PAGE and western blotting

Usually, total protein extracts corresponding to 0.0625 OD_600_ units of cells per sample were separated on 12.5% SDS-PAA gels. For detection of Rne_NTD_-FLAG 8% SDS-PAA gels were used. Proteins were either visualized by Coomassie Brilliant Blue R-250 staining or transferred to a PVDF membrane (Amersham) via semi-dry blotting (Peqlab) for 90–120 min at 120 mA. The membranes were incubated overnight at 4°C with the desired primary antiserum (α-FLAG diluted 1:25,000, antibodies-online; GlmS antiserum 1:10,000; S2 antiserum diluted 1:5000). Signal detection was achieved by incubation with anti-rabbit IgG AP-conjugated secondary antibodies (Promega) diluted 1:100,000 and using CDP* (Roche Diagnostics) as substrate.

### RNA extraction and northern blotting

Total RNA was extracted using the ReliaPrep RNA Cell Miniprep System (Promega). Usually, 5 µg total RNA or designated volumes of the RNA solutions derived from ligand fishing experiments (normalized to protein content) were mixed with 2× RNA loading dye (95% formamide, 0.5 mM EDTA, 0.025% SDS, 0.025% bromophenol blue and 0.025% xylene-cyanol) and separated on denaturing gels (7 M urea, 6% acrylamide, 1× TBE) using 0.5× TBE as running buffer. RNA was transferred to a positively charged nylon membrane (Hybond^+^, GE Healthcare) by electroblotting (1 h, 120 mA) using 0.5× TBE as transfer buffer and crosslinked by UV radiation. For detection of longer transcripts, 7.5 µg total RNA was separated by formaldehyde agarose gel electrophoresis (1% agarose, 18% formaldehyde, 20 mM MOPS, 5 mM sodium acetate, 1 mM EDTA, pH 7.0) and transferred to Hybond^+^ nylon membrane via vacuum blotting (VacuGene XL, Amersham Biosciences) at 85 mbar for 4 h. To obtain loading controls, 23S and 16S rRNAs were visualized by ethidium bromide staining before transfer. Probes were synthesized and labeled with digoxigenin (DIG) by in vitro transcription using T7 RNA polymerase, the DIG RNA labeling mix (Roche Diagnostics) and PCR-generated DNA templates. Primers to generate the PCR templates were BG230/BG231 (*glmZ*), BG260/BG261 (*glmY*), BG149/BG150 (*glmS*), BG1915/BG1916 (*entC*), BG1911/BG1912 (*cirA*), BG1917/BG1918 (*malQ*), BG1942/BG1943 (*cspB*), BG1927/BG1928 (*cspH*), BG1919/BG1920 (*fimA*), and BG287/BG288 (*rrfD*). Detection of specific signals was achieved by incubation with anti-DIG AP-conjugated antibodies and CDP* as substrate (Roche Diagnostics).

### Purification of recombinant proteins

Purification of Strep-tagged proteins and His-tagged RNase E is described under Supplemental Material.

### In vitro transcription and labeling of RNA

Transcription and radioactive labeling of RNA in vitro was performed as described recently (Durica-Mitic and Görke 2019). For generation of the required DNA templates, the following oligonucleotides were used: BG444/BG445 for GlmZ, BG1684/BG1685 for *uspF*, BG1686/BG1250 for *rpsT* and BG1511/BG1512 for *glyX–glyY*.

### EMSA

EMSA was performed as described previously (Göpel et al. 2016; Durica-Mitic and Görke 2019).

### In vitro pull-down assay using Ni-NTA magnetic agarose beads

Purified His_10_-Rne_NTD_ (3.5 µg) was coupled to 12.5 µL magnetic Ni-NTA beads (Qiagen) by incubation in 250 µL interaction buffer (50 mM NaH_2_PO_4_, 300 mM NaCl, 10 mM MgCl_2_, 40 mM imidazole, 0.005% Tween 20, pH = 8.0) for 1 h at 4°C. Subsequently, the supernatant was removed and 3.5 µg of the candidate protein provided in 250 µL interaction buffer was added. Following an additional incubation at RT for 1.5 h, beads were 4× washed using 500 µL interaction buffer each. For analysis of the bound protein fraction, the beads were dissolved in 50 µL Laemmli buffer and 10 µL were separated by SDS-PAGE followed by Coomassie blue silver staining (Candiano et al. 2004). For RNA removal, purified Strep-RapZ and His_6_-Rne_NTD_ were preincubated with 600 µ micrococcal nuclease (NEB) in 1× reaction buffer for 1 h at 37°C and subsequently used for in vitro pull-down assay.

### RNase E cleavage assay

RNase E cleavage assays were carried out in 1× reaction buffer (25 mM Tris-HCl pH 7.5, 50 mM NaCl, 10 mM MgCl_2_, and 1 mM DTT) in 10 µL volume, as described previously (Göpel et al. 2013; Durica-Mitic and Görke 2019). α-^32^P-UTP labeled GlmZ was mixed with 1 µg yeast tRNA (Ambion), heat-denatured, chilled and incubated for 5 min at 30°C. Subsequently, required amounts of the respective RapZ variant were added and following an additional incubation for 10 min, the assay was started by adding Rne_NTD_ (usually 50 nM). In end point assays, reactions were stopped following the indicated incubation time (usually 40 min) by addition of 0.2 U of proteinase K (NEB) and further incubation for 30 min at 50°C. In the case of time course analyses, assay volumes were scaled up accordingly and 10 µL aliquots were removed at indicated times to stop reactions by proteinase K treatment. Finally, samples were mixed with 2× RNA loading dye (95% formamide, 0.5 mM EDTA, 0.025% SDS, 0.025% bromophenol blue, 0.025% xylene-cyanol) and separated on denaturing polyacrylamide gels (7 M urea, 6% acrylamide, 1× TBE) using 1× TBE as running buffer. Gels were analyzed by phospho-imaging except for assays using cold GlmZ RNA, which were visualized by ethidium bromide (0.5 mg/mL) staining. Cleavage assays using *glyX–glyY* RNA were performed at 37°C.

### Whole transcriptome analysis by RNA-sequencing

The whole transcriptome analysis is described under Supplemental Material.

## SUPPLEMENTAL MATERIAL

Supplemental material is available for this article.

## Supplementary Material

Supplemental Material
