# Supplementary material for: Adaptor protein RapZ activates endoribonuclease RNase E by protein–protein interaction to cleave a small regulatory RNA
Source: RNA. 2020 Sep;26(9):1198–215. doi: 10.1261/rna.074047.119 (PMC7430671; doi:10.1261/rna.074047.119)
Supplement: Supplemental Material [file supp_26_9_1198__index.html]

Adaptor protein RapZ activates endoribonuclease RNase E by protein–protein interaction to cleave a small regulatory RNA — Supplemental Material 

# Adaptor protein RapZ activates endoribonuclease RNase E by protein–protein interaction to cleave a small regulatory RNA

## Supplemental Material

- Supplemental\_Legends.docx
- Supplemental\_Material.docx
- Supplemental\_Table\_S3.xlsx
- Supplemental\_Table\_S4.xlsx
- Supplemental\_Table\_S6.xlsx
- Supplemental\_Table\_S7.xlsx
