# Supplementary material for: Adaptor protein RapZ activates endoribonuclease RNase E by protein–protein interaction to cleave a small regulatory RNA
Source: RNA. 2020 Sep;26(9):1198–215. doi: 10.1261/rna.074047.119 (PMC7430671; doi:10.1261/rna.074047.119)
Supplement: Supplemental Material [file supp_074047.119_Supplemental_Legends.docx]

**SUPPLEMENTAL MATERIAL**

Files:

**Supplemental material (.docx)** contains:

- Supplemental Figures S1 – S9
- Supplemental Tables S1, S2, S5, S8, S9, S10, S11
- Supplemental Materials and Methods
  - Construction of plasmids
  - Purification of Strep-tagged proteins
  - Purification of His-tagged RNase E-NTD (aa 1-529)
  - Whole transcriptome analysis by RNA-sequencing
- Supplemental References

**Supplemental Table S3 (xlsx).** Source data including statistical analysis for the β-galactosidase activity assays presented in Figs. 1 and 2.

**Supplemental Table S4 (xlsx).** Complete list of RNA-seq results comparing strains Z37 (*ΔrapZ*), Z864 (*ΔrapZ ΔglmY ΔglmZ*) and Z106 (*ΔglmY ΔglmZ*) with R1279 (*wild type*), respectively.

**Supplemental Table S6 (xlsx).** List of RNA-seq results comparing strain Z37 (*ΔrapZ*) with Z37 pBGG237 (*ΔrapZ* + empty plasmid), Z37 pBGG164 (*ΔrapZ* + *rapZ* on plasmid) and Z37 pYG29 (*ΔrapZ* + *rapZ_quad_* on plasmid), respectively. Moreover, RNA-seq results are included comparing strain Z864 (*ΔrapZ ΔglmY ΔglmZ*) with Z864 pBGG237 (*ΔrapZ* *ΔglmY ΔglmZ* + empty plasmid) and Z864 pBGG164 (*ΔrapZ* *ΔglmY ΔglmZ* + *rapZ* on plasmid), respectively.

**Supplemental Table S7 (xlsx).** List of putative direct entry RNase E substrates obtained by mapping the cleavage sites identified in (Clarke et al. 2014) to the genome.
