# Supplementary material for: Adaptor protein RapZ activates endoribonuclease RNase E by protein–protein interaction to cleave a small regulatory RNA
Source: RNA. 2020 Sep;26(9):1198–215. doi: 10.1261/rna.074047.119 (PMC7430671; doi:10.1261/rna.074047.119)
Supplement: Supplemental Material [file supp_074047.119_Supplemental_Material.docx]

**SUPPLEMENTAL MATERIAL for**

**Adaptor protein RapZ activates endoribonuclease RNase E by protein-protein interaction to cleave a small regulatory RNA**

Svetlana Durica-Mitic^1^, Yvonne Göpel^1^, Fabian Amman^2,3^ and Boris Görke^1*^

^1^Department of Microbiology, Immunobiology and Genetics, Max Perutz Labs, University of Vienna, Vienna Biocenter (VBC), Vienna, Austria.

^2^Center for Anatomy and Cell Biology, Medical University of Vienna, Vienna, Austria.

^3^Institute of Theoretical Biochemistry, University of Vienna, Vienna, Austria.

**This file contains:**

- Supplemental Figures S1 – S9
- Supplemental Tables S1, S2, S5, S8, S9, S10, S11
- Supplemental Materials and Methods
  - Construction of plasmids
  - Purification of Strep-tagged proteins
  - Purification of His-tagged RNase E-NTD (aa 1-529)
  - Whole transcriptome analysis by RNA-sequencing
- Supplemental References

**SUPPLEMENTAL FIGURES**


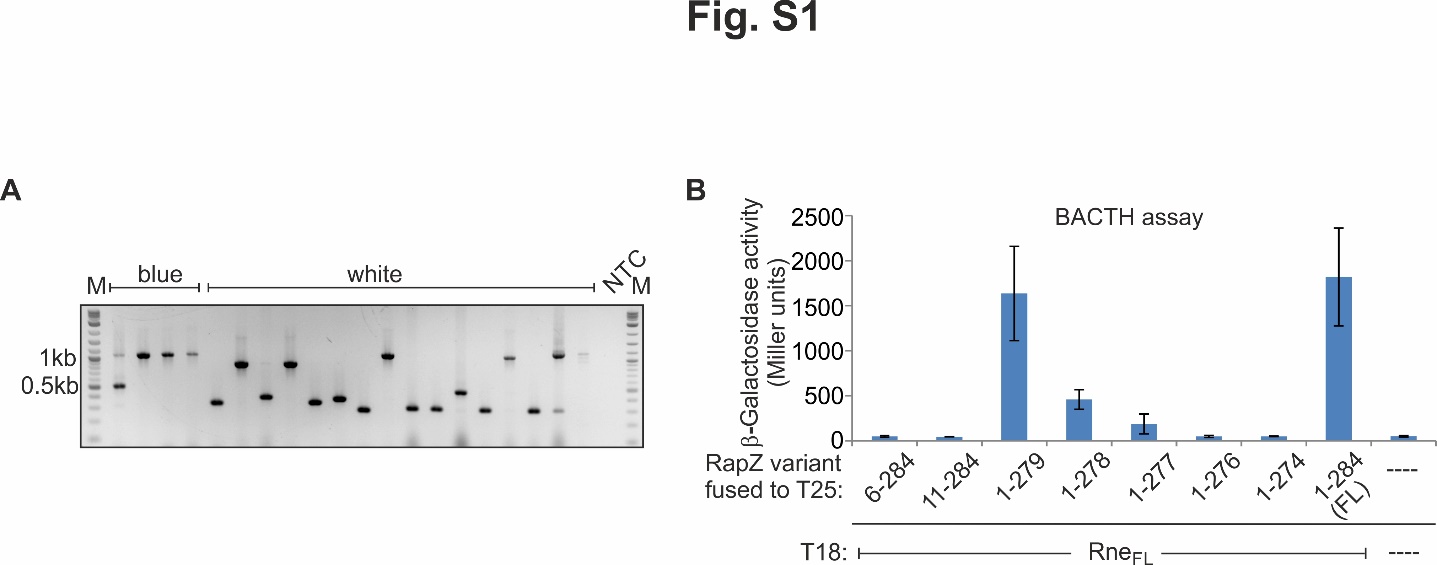


**Supplemental Figure S1. RNase E interaction potential of truncated RapZ variants. (*A*)** The *rapZ* inserts of blue as well as white recombinants obtained in the BACTH screen were PCR-amplified and fragment sizes were determined by 1% agarose gel electrophoresis. NTC = no template control. **(*B*)** BACTH analysis of interaction of defined RapZ truncations (fused to T25) with T18-Rne_FL_ (encoded on plasmid pYG99). Similar analysis as shown in Fig. 1C but T25- and T18-domains were swapped between the candidate proteins. The following plasmids encoding fusions of RapZ variants to T25 were used: pSD116 (RapZ_6-284_), pSD117 (RapZ_11-284_), pSD118 (RapZ_1-279_), pSD142 (RapZ_1-278_), pSD143 (RapZ_1-277_), pSD132 (RapZ_1-276_), pSD119 (RapZ_1-274_), pBGG348 (RapZ_FL_), pKT25 (no fusion to T25).


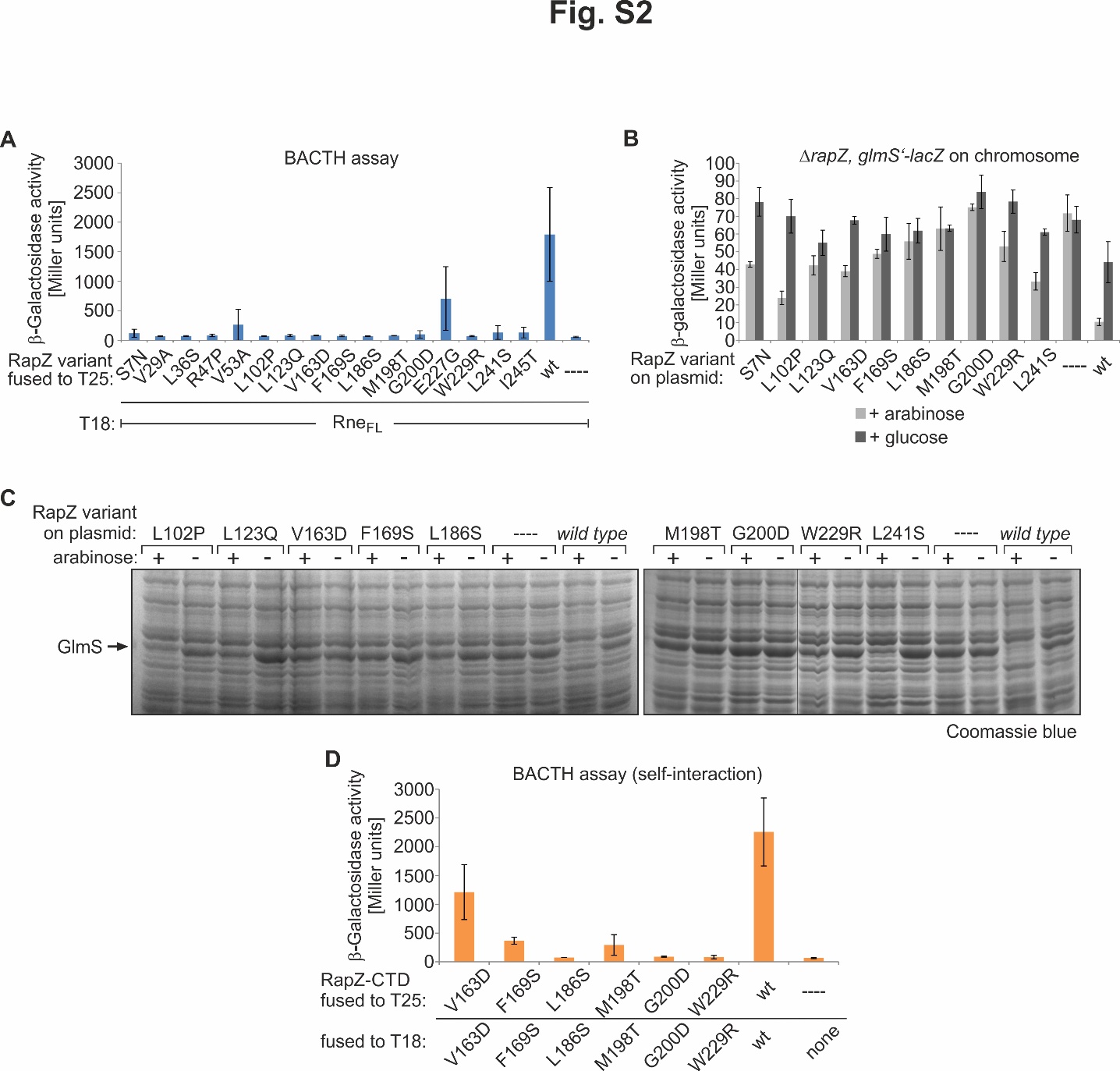


**Supplemental Figure S2. Analysis of RapZ mutants obtained in the random mutagenesis screen for decreased interaction with RNase E. (*A*)** BACTH analysis of interaction of the screen-derived RapZ mutants fused to T25 (listed in Supplemental Table S1) with Rne_FL_ fused to T18 encoded on plasmid pYG99. **(*B*)** Ability of plasmid-encoded RapZ mutants to repress *glmS* expression in strain Z28, which lacks endogenous *rapZ* and harbors an ectopic *glmS’-lacZ* fusion on the chromosome. Expression of the *rapZ* mutants was driven from the arabinose-inducible *P_ara_* promoter (plasmids are listed in Supplemental Table S10). Empty plasmid pBAD33 and plasmid pBGG61 encoding *wild type* RapZ served as negative and positive controls, respectively (penultimate and ultimate columns). Expression of *rapZ* was either induced with 0.2% arabinose or repressed by 0.1% glucose. β-Galactosidase activities were determined from exponentially growing cells. **(*C*)** Evaluation of GlmS protein levels in the cells assessed in (*B*). Total protein extracts corresponding to 0.0625 OD_600_ units of cells were separated on 10% SDS-PAA gels and stained with Coomassie blue. Previous work has shown that *ΔrapZ* mutants accumulate GlmS to levels visible by eye in such gels (Kalamorz et al. 2007; Gonzalez et al. 2017). **(*D*)** BACTH assay addressing dimerization of the RapZ_CTD_ carrying substitutions detected in the random mutagenesis screen. Plasmids pSD10 and pSD12 encoding *wild type* T25-RapZ_CTD_ and T18-RapZ_CTD_, respectively, served as the positive control. The negative control is provided by plasmids pKT25/pUT18C. Plasmids encoding the mutant T25- and T18-RapZ_CTD_ variants are listed in Supplemental Table S10 (pSD39-pSD78). Presented values derive from a minimum of two measurements.


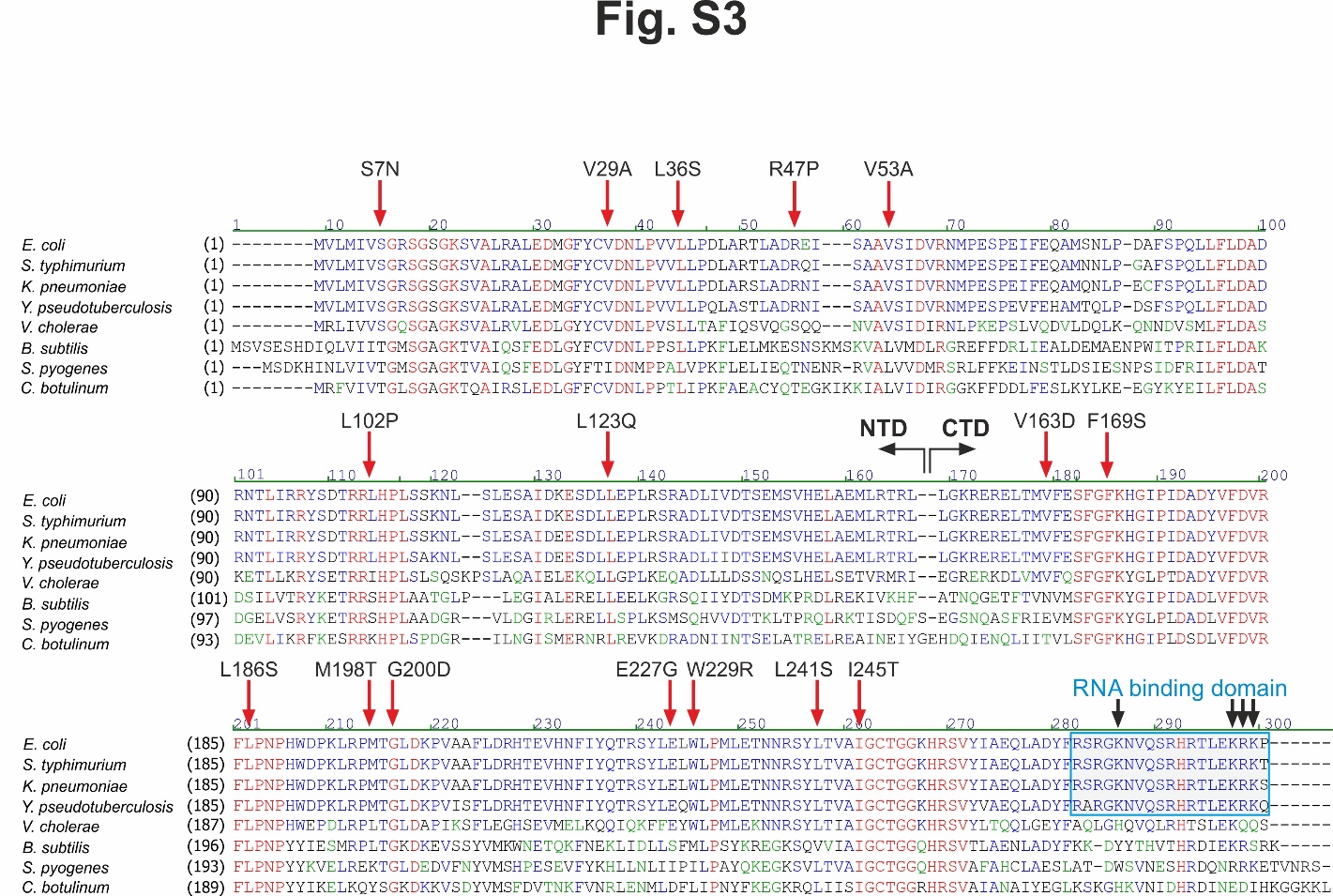


**Supplemental Figure S3. Location of the amino acid substitutions identified in the random mutagenesis screen for RapZ variants impaired in interaction with RNase E.** Alignment of RapZ homologs of various bacteria. Positions of single amino acid substitutions identified in the random mutagenesis screen of RapZ are marked with red arrows. The position separating the NTD and CTD is indicated by orthogonal arrows. The C-terminal RNA-binding domain composed of multiple positively charged residues is boxed in blue. Black arrows indicate the residues which are replaced with alanine in the RapZ_quad_ mutant, abrogating RNA-binding activity. Note that the RNA-binding domain is only conserved in those bacteria i.e. in *Enterobacteriaceae*, which also possess the GlmY/GlmZ sRNAs.

**Fig. S4**

**
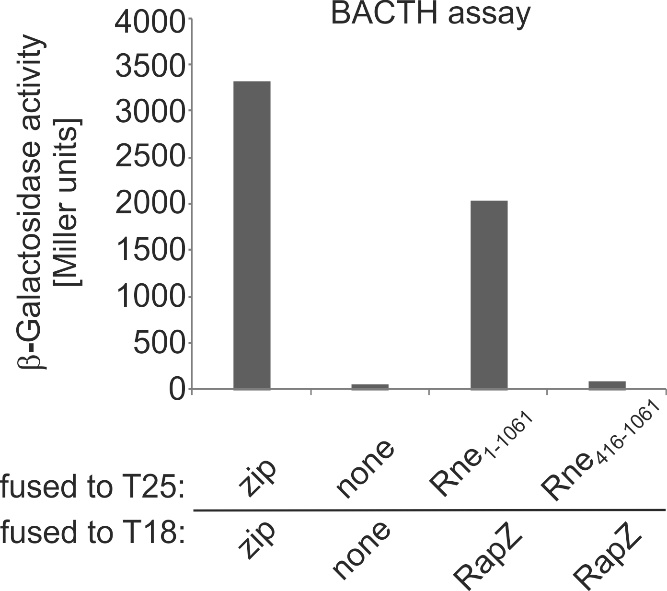
**

**Supplemental Figure S4. Preliminary BACTH analysis addressing interaction of RapZ with an RNase E variant (Rne_416-1061_) lacking the large globular domain.** The following T25 encoding plasmids were used (from left to right): pKT25-zip, pKT25, pYG100 and pSD191. The T18 encoding plasmids were pUT18-zip, pUT18C, and pBGG349. Please note that due to working restrictions at the time of revision work, only one biological replicate could be tested. However, measurements are in agreement with corresponding colony phenotypes observed on X-Gal plates, confirming the result.

**Fig. S5**


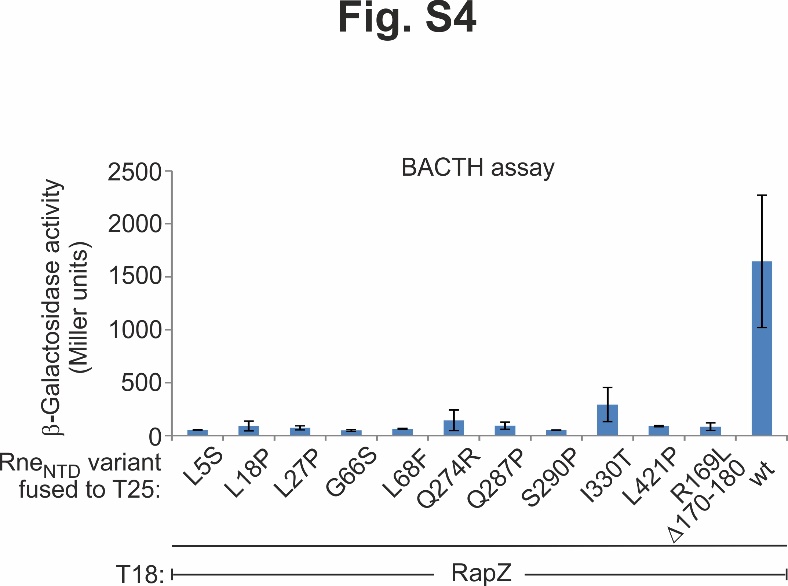


**Supplemental Figure S5. Quantitative BACTH analysis of the Rne_NTD_ mutants derived from the random mutagenesis screen for abrogated interaction with RapZ.** BACTH assay addressing interaction of screen-derived Rne_NTD_ variants fused to T25 (listed in Supplemental Table S2) with T18-RapZ encoded on plasmid pBGG349. Plasmid pYG101 encoding *wild type* T25-Rne_NTD_ was included for comparison (last column). Presented values derive from a minimum of two measurements.

**Fig. S6**


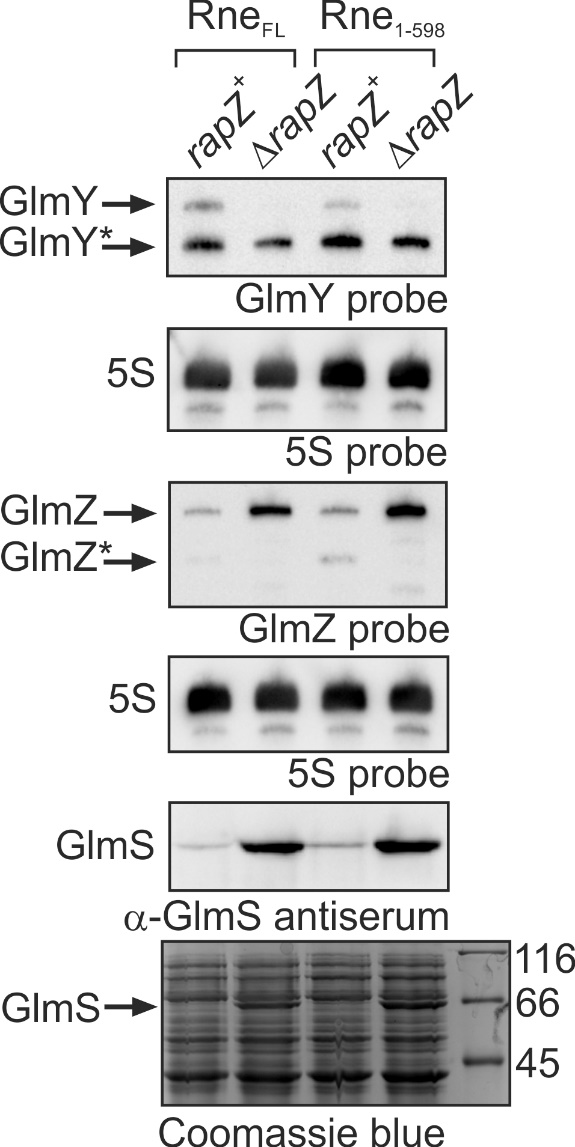


**Supplemental Figure S6. The C-terminal scaffolding domain of RNase E does not play a role in the regulatory GlmY/GlmZ/*glmS* circuit.** GlmY, GlmZ and GlmS levels were assessed in strains Z64 (*rne-FLAG*, lane 1), Z1242 (*rne-FLAG* *ΔrapZ*; lane 2), Z1241 (*rne_1-598_-FLAG*, lane 3) and Z903 (*rne_1-98_-FLAG* *ΔrapZ*; lane 4). Bacteria were grown to exponential phase and samples were harvested for isolation of total RNA and protein. Total RNA was analyzed by Northern blotting for detection of GlmY (top panel), GlmZ and 5S rRNA (loading control). Total protein extracts were separated on SDS-PAA gels and subjected to Western blotting using α-GlmS antiserum (penultimate panel) or stained with Coomassie blue (bottom panel).

**Fig. S7**

**
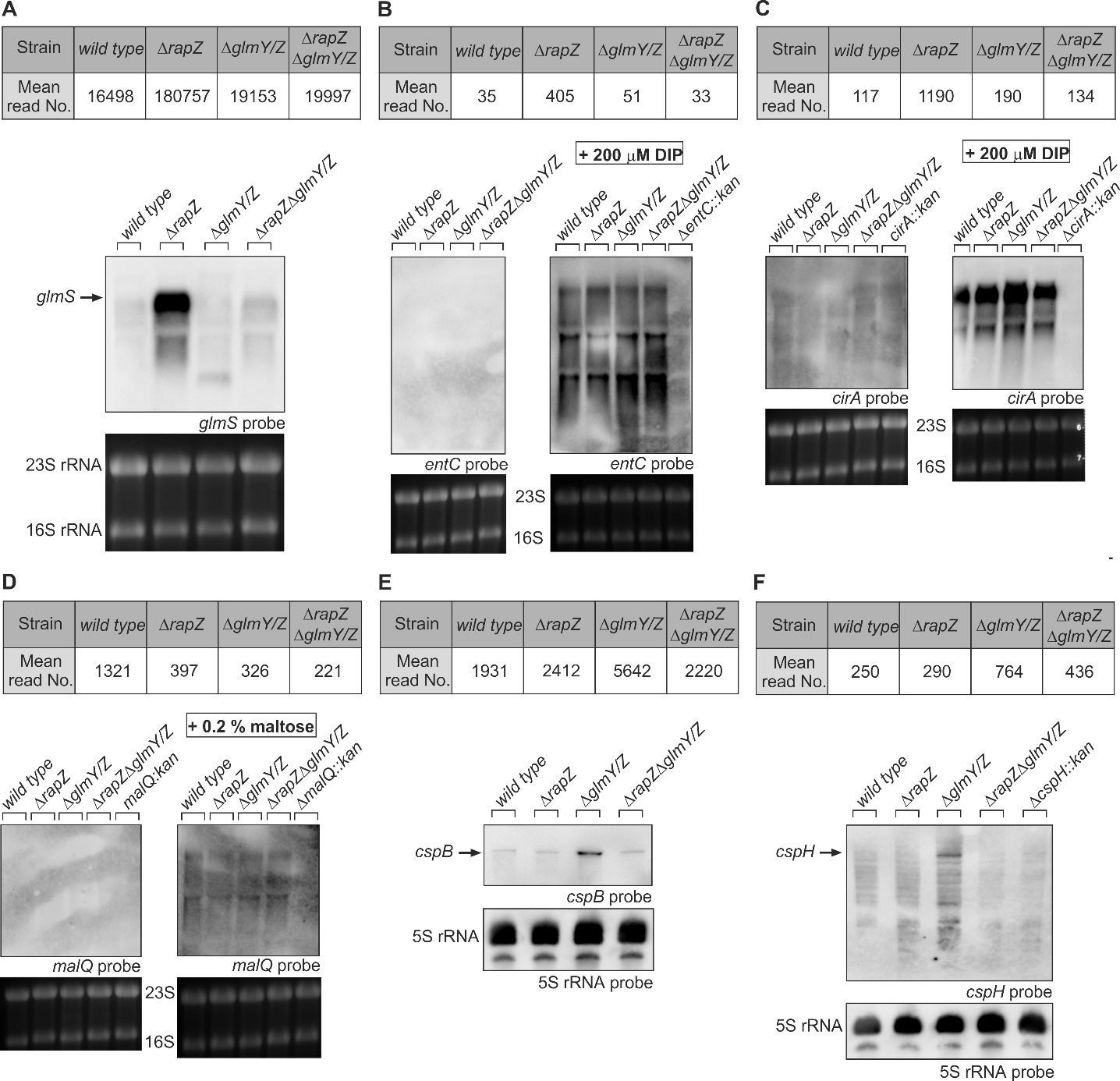
**

**Supplemental Figure S7. Northern blot analyses for validation of RNA-seq results.** Strains R1279 (*wild type*), Z37 (Δ*rapZ*), Z106 (Δ*glmY* Δ*glmZ*) and Z864 (Δ*rapZ* Δ*glmY* Δ*glmZ*) were grown to OD_600_ ~ 0.3. Subsequently, total RNAs were isolated and assessed by Northern blotting using probes specific for *glmS* **(*A*),** *entC* **(*B*),** *cirA* **(*C*),** *malQ* **(*D*)**, *cspB* **(*E*)** and *cspH* **(*F*).** Where appropriate, strains were grown in presence of 200 μM DIP or 0.2% maltose to induce expression of iron and maltose utilization genes, respectively. DIP was added when cultures reached OD_600_ ~ 0.2 and cells were harvested 30 min later. Where available, deletion strains were included to confirm specificity of the probes. Loading controls are provided by 23S/16S rRNA or 5S rRNA signals. Tables above the blots report the corresponding mean read numbers obtained by RNA-seq.

**Fig. S8**


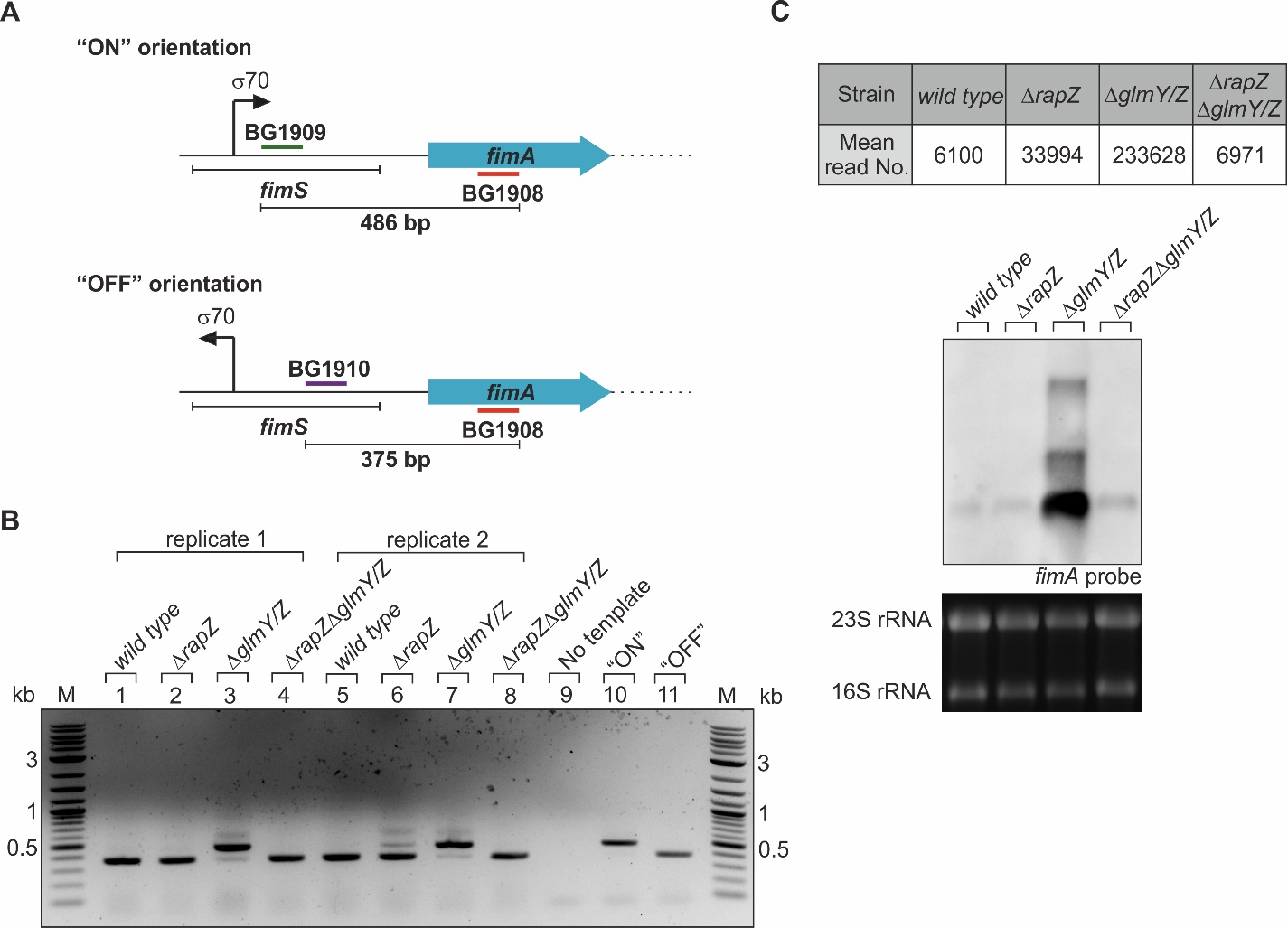


**Supplemental Figure S8. Analysis of the *fimS*/*fimA* locus in strains subjected to RNA-seq. (*A*)** Schematic representation of the diagnostic PCR approach addressing orientation of the *fimA* promoter contained within the invertible *fimS* switch. Two forward primers, each specific for a different *fimS* orientation, are used together with the common reverse primer BG1908 in PCR. Primer combination BG1909/1908 is specific for the “ON” orientation and generates a 486 bp fragment (top). In contrast, primers BG1910/BG1908 yield a 375 bp PCR product when the *fimS* switch is in the “OFF” orientation (bottom). **(*B*)** PCR analysis of *fimS* orientation in the RNA samples that were subjected to RNA-seq. Aliquots of the RNA samples were collected prior to the DNA removal step with the Turbo DNA-free kit and therefore contained genomic DNA contaminations, which served as templates for PCR. Each reaction contained the required RNA sample (volume corresponding to 300 ng RNA) and the three primers BG1908, BG1909, BG1910. Following 30 amplification cycles, reactions were separated by 1% agarose gel electrophoresis. Control fragments diagnostic for the “ON” and “OFF” states, respectively, served as additional size markers (lanes 10 and 11) and were generated by PCRs using only one of the two forward primers (i.e. BG1909/BG1908 for “ON” and BG1910/BG1908 for “OFF” orientation of *fimS*). A control PCR containing no template (RNA sample) was included in lane 9. The ethidium bromide stained agarose gel is shown. The results show that the *fimS* switch was in the “OFF” orientation in the samples derived from the *wild type*, the *ΔrapZ* *ΔglmY ΔglmZ* triple mutant and replicate 1 of the *ΔrapZ* mutant. In contrast, a faint band for the fragment indicative of the “ON” orientation was detectable in replicate 2 of the *ΔrapZ* mutant and this fragment predominated in both replicates of the *ΔglmY ΔglmZ* double mutant. Thus, *fimS* switched into the “ON” orientation in a minority (*ΔrapZ*, replicate 2) or majority (*ΔglmY ΔglmZ*, both replicates) of cells analyzed by RNA-seq. **(*C*)** Northern analysis investigating *fimA* abundance in the original strain isolates used to inoculate the cultures analyzed by RNA-seq. Strains R1279 (*wild type*), Z37 (Δ*rapZ*), Z106 (Δ*glmY* Δ*glmZ*) and Z864 (Δ*rapZ* Δ*glmY* Δ*glmZ*) were grown to OD_600_ ~ 0.3 and samples were harvested for extraction of total RNA and Northern analysis of the *fimA* transcript. The results show that *fimA* is strongly up-regulated in the *ΔglmY ΔglmZ* strain correlating with the mean read numbers obtained by RNA-seq. This indicates that the switch into the *fimS* “ON” orientation might have happened during construction of the strain. In contrast, no up-regulation of *fimA* was detected in the *ΔrapZ* mutant suggesting that the switch observed in (B) happened during growth of the culture (*ΔrapZ*, replicate 2) for RNA-seq, resulting in only a fraction of cells carrying the *fimS* “ON” orientation.

**Fig. S9**

**
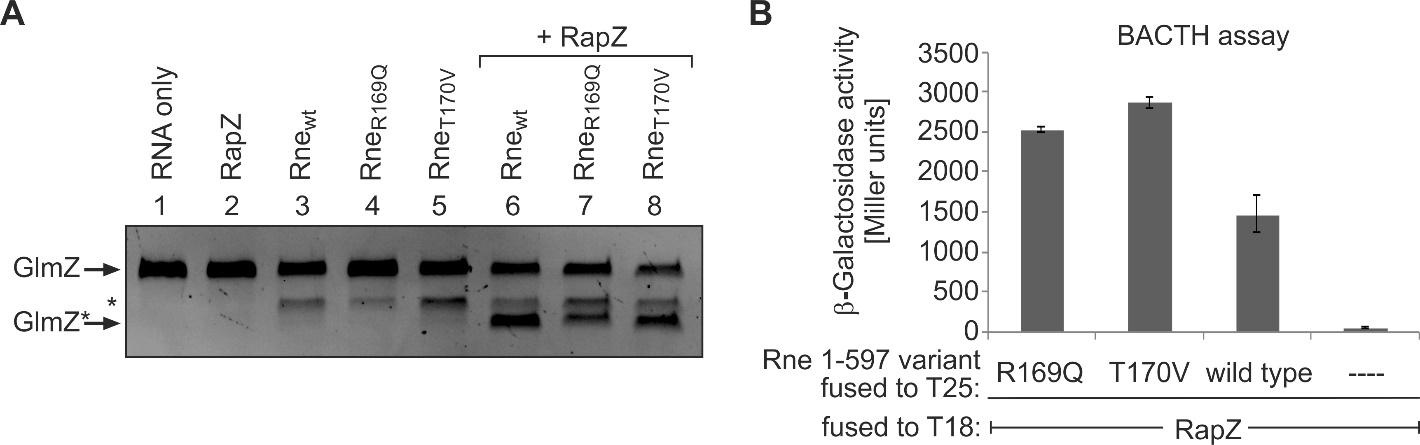
**

**Supplemental Figure S9. RNase E variants impaired in 5’ monophosphate sensing retain the ability to interact with RapZ and to cleave GlmZ in a RapZ-dependent manner *in vitro*. (*A*)** “Cold” *in vitro* cleavage assay comparing cleavage of GlmZ by wild type Rne_NTD_, Rne_NTD_-R169Q and Rne_NTD_-T170V in absence or presence of RapZ. 50 nM unlabeled (“cold”) GlmZ was incubated with 300 nM of the indicated Rne_NTD_ variant in the absence (lanes 3-5) or presence of 1200 nM RapZ (lanes 6-8) for 30 min. Additionally, GlmZ was incubated alone (lane 1) or with RapZ only (lane 2). Reactions were separated on denaturing PAA gels and visualized by ethidium bromide staining. An unspecific cleavage product is indicated with an asterisk. **(*B*)** BACTH assay addressing interaction of T18-RapZ (encoded on plasmid pBGG349) with T25-Rne_NTD_ variants encoded on plasmid pSD18 (Rne_NTD_-R169Q), pSD19 (Rne_NTD_-T170V) and pYG101 (wild type Rne_NTD_).

**SUPPLEMENTAL TABLES**

**Supplemental Table S1**. Single amino acid substitutions in RapZ identified in the random mutagenesis screen for loss of interaction with RNase E.

| **Amino acid substitution** | **Nucleotide exchange** | **Number found** | **Isolate name** |
| --- | --- | --- | --- |
| Ser7 → Asn | AGC → AAC | 2 × | RapZ 1.4, RapZ 1.6 |
| Val29 → Ala | GTG → GCG | 1 × | RapZ 1.10 |
| Leu36 → Ser | TTG → TCG | 2 × | RapZ 1.1, RapZ 1.9 |
| Arg47 → Pro | CGC → CCC | 1 × | RapZ 2.3 |
| Val53 → Ala | GTC → GCC | 1 × | RapZ 2.9 |
| Leu102 → Pro | CTG → CCG | 1 × | RapZ 4.3 |
| Leu123 → Gln | CTG → CAG | 1 × | RapZ 1.5 |
| Val163 → Asp | GTC → GAC | 1 × | RapZ 5.6 |
| Phe169 → Ser | TTC → TCC | 2 × | RapZ 2.2, RapZ 2.4 |
| Leu186 → Ser | TTG → TCG | 1 × | RapZ 2.5 |
| Met198 → Thr | ATG → ACG | 1 × | RapZ 1.3 |
| Gly200→ Asp | GGT → GAT | 1 × | RapZ 2.6 |
| Glu227 → Gly | GAG → GGG | 1 × | RapZ 4.1 |
| Trp229 → Arg | TGG → CGG | 1 × | RapZ 2.10 |
| Leu241 → Ser | TTG → TCG | 1 × | RapZ 1.8 |
| Ile245 → Thr | ATT → ACT | 1 × | RapZ 1.2 |

*The RapZ Val163Asp mutant additionally contains a silent mutation in lysine codon 155 (AAA→AAG)

**Supplemental Table S2**. Rne_NTD_ mutants identified in the random mutagenesis screen for impaired interaction with RapZ.

| **Amino acid substitution/deletion** | **Nucleotide exchange/deletion** | **Number found** | | | **Isolate name** |
| --- | --- | --- | --- | --- | --- |
| **A. Single amino acid substitutions** | | | | | |
| Leu5 → Ser | TTA → TCA | | 1 × | | Rne 3-20 |
| Leu18 → Pro | CTT → CCT | | 2 × | | Rne1-27, Rne3-4 |
| Leu27 → Pro | CTG → CCG | | 3 × | | Rne 3-17, Rne 3-48, Rne 3-41 |
| Gly66 → Ser | GGT → AGT | | 1 × | | Rne 1-38 |
| Leu68 → Phe, Ser227 → Ser (silent), Cys471 → Cys (silent) | CTC → TTC, TCC → TCT, TGT → TGC | | 1 × | | Rne 1-1 |
| Gln274 → Arg | CAG → CGG | | 1 × | | Rne 2-10W |
| Ser287 → Pro | TCT → CCT | | 1 × | | Rne 1-31 |
| Ser290 → Pro | TCC → CCC | | 4 × | | Rne 3-38, Rne 3-37, Rne 3-43, Rne 3-44 |
| Ile330 → Thr | ATT → ACT | | 1 × | | Rne 2-4 |
| Leu421 → Pro | CTC → CCC | | 1 × | | Rne 1-21 |
| **B. Multiple amino acid substitutions** | | | | | |
| Ile41 → Thr, Arg332 → Arg (silent), Cys407 → Arg | ATC → ACC, CGT → CGC, TGT → CGT | | 1 × | | Rne 3-21 |
| Gln103 → Arg, Gln242 → Arg, Arg414 → His | CAG → CGG, CAG → CGG, CGT → CAT | | 1 × | | Rne 3-35 |
| Gly261 → Ser, Leu285 → Pro | GGC → AGC, CTG → CCG | | 1 × | | Rne 1-30 |
| Val403 → Val (silent), Ser422 → Ser (silent), Ile473 → Val, Val474 → Ala | GTT → GTC, TCT → TCC, ATT → GTT, GTG → GCG | | 1 × | | Rne 3-31 |
| **C. Amino acid substitutions + in frame deletion** | | | | | |
| Arg75 → Ser, Arg169 → Leu, Δ170-180 | CGC → AGC, CGC → CTG, Δ506-538 | | | 1 × | Rne 1-12 |
| Arg169 → Leu, Δ170-180 | CGC → CTG, Δ506-538 | | | 1 × | Rne 1-14 |

**Supplemental Table S5.** Genes that are differentially regulated in the *ΔglmY* *ΔglmZ* double mutant as compared to the *wild type* strain (strain Z106 vs. R1279).

| **Gene name** | **log2 fold change** | **adjusted**  **p-value** | **mean normalized reads in *ΔglmY ΔglmZ* strain** | **mean normalized reads in *wild type* strain** |
| --- | --- | --- | --- | --- |
| *leuE* | 6.416 | 3.7e-21 | 244.3 | 2.8 |
| *fimA* | 5.259 | 1.21e-13 | 233628.2 | 6100.2 |
| *fimC* | 5.189 | 4.19e-13 | 10360.8 | 284.1 |
| *fimG* | 5.083 | 4.81e-18 | 1509.9 | 44.7 |
| *fimI* | 5.059 | 3.98e-20 | 15590.8 | 467.8 |
| *fimD* | 4.953 | 3.81e-19 | 12883.1 | 416.1 |
| *fimF* | 4.925 | 6.03e-19 | 1860.7 | 61.1 |
| *fimH* | 4.856 | 4.06e-10 | 3569.3 | 123.4 |
| *ybaA* | 2.027 | 0.00286 | 188.4 | 47.2 |
| *ibpB* | 1.887 | 0.0181 | 269.2 | 72.4 |
| *cspI* | 1.815 | 2.77e-07 | 190.5 | 55.6 |
| *ydfK* | 1.786 | 0.0167 | 46.1 | 13.4 |
| *cspH* | 1.614 | 0.0436 | 763.8 | 250.1 |
| *cspB* | 1.548 | 2.13e-06 | 5642.2 | 1930.9 |
| *cspF* | 1.548 | 0.000723 | 809.4 | 278.2 |
| *aslB* | 1.498 | 0.0181 | 220.7 | 79.3 |
| *ynaE* | 1.474 | 0.00413 | 98.2 | 36.4 |
| *treC* | 1.402 | 0.0263 | 465.8 | 176.0 |
| *cspG* | 1.279 | 0.000106 | 6179.9 | 2548.1 |
| *yghF* | -1.233 | 0.0263 | 60.9 | 144.3 |
| *yghG* | -1.245 | 0.0504 | 39.9 | 95.5 |
| *stpA* | -1.440 | 4.64e-05 | 706.7 | 1917.2 |
| *flgF* | -1.505 | 0.0308 | 14.2 | 38.7 |
| *fliF* | -1.575 | 0.0129 | 17.0 | 48.2 |
| *fliL* | -1.575 | 0.0398 | 9.2 | 26.0 |
| *yghJ* | -1.624 | 4.3e-14 | 732.1 | 2256.7 |
| *flgB* | -1.734 | 0.0201 | 9.1 | 29.2 |
| *flgD* | -1.737 | 0.0181 | 15.7 | 50.4 |
| *fliA* | -2.644 | 1.9e-06 | 7.8 | 47.3 |
| *glmZ* | -5.030 | 1.06e-09 | 80.6 | 2632.9 |
| *glmY* | -6.655 | 2.03e-06 | 0.9 | 87.1 |

**Supplemental Table S8.** List of the 36 genes that are differentially regulated in all three datasets presented in Fig. 6D and Supplemental Table S6 (|log2 fold change| ≥ 1.0; adjusted p-value ≤ 0.05). Genes whose transcripts were previously identified as putative substrates for RNase E (Deana et al. 2008; Clarke et al. 2014) are indicated with “X”.

| **gene** | **log2 fold change**  ***ΔrapZ* strain**  **compared to wild type RapZ overproduction** | **log2 fold change**  ***ΔrapZ* strain upon RapZ_quad_ overproduction** | **log2 fold change *ΔrapZ ΔglmY* *ΔglmZ* strain upon wild type RapZ overproduction** | **Present in dataset from (Deana et al. 2008)** | **Present in dataset from (Clarke et al. 2014)** | **Functional annotation** |
| --- | --- | --- | --- | --- | --- | --- |
| [*argH*](https://www.biocyc.org/ECOLI/substring-search?type=GENE&object=argH&geneSearch=Gene) | -1.43 | 1.117 | -1.937 |  |  | argininosuccinate lyase |
| [*cysZ*](https://www.biocyc.org/ECOLI/substring-search?type=GENE&object=cysZ&geneSearch=Gene) | 1.41 | 1.109 | 1.3 |  |  | sulfate transporter |
| [*dsbA*](https://www.biocyc.org/ECOLI/substring-search?type=GENE&object=dsbA&geneSearch=Gene) | 1.767 | 1.387 | 1.128 |  |  | thiol:disulfide oxidoreductase |
| [*eda*](https://www.biocyc.org/ECOLI/substring-search?type=GENE&object=eda&geneSearch=Gene) | 2.088 | 1.481 | 1.203 |  |  | KHG/KDPG aldolase |
| [*fabI*](https://www.biocyc.org/ECOLI/substring-search?type=GENE&object=fabI&geneSearch=Gene) | 2.514 | 1.719 | 1.362 | X |  | enoyl-[acyl-carrier-protein] reductase |
| [*fecA*](https://www.biocyc.org/ECOLI/substring-search?type=GENE&object=fecA&geneSearch=Gene) | 1.358 | 1.122 | 1.066 |  |  | ferric citrate transporter |
| [*fldA*](https://www.biocyc.org/ECOLI/substring-search?type=GENE&object=fldA&geneSearch=Gene) | 1.677 | 1.132 | 1.045 | X |  | flavodoxin 1 |
| [*fryC*](https://www.biocyc.org/ECOLI/substring-search?type=GENE&object=fryC&geneSearch=Gene) | 2.538 | 2.041 | 1.177 |  |  | putative PTS component |
| [*ftnA*](https://www.biocyc.org/ECOLI/substring-search?type=GENE&object=ftnA&geneSearch=Gene) | 2.546 | 1.158 | 1.662 |  |  | ferritin iron-storage complex |
| [*galP*](https://www.biocyc.org/ECOLI/substring-search?type=GENE&object=galP&geneSearch=Gene) | 3.402 | 2.315 | 1.471 |  |  | galactose:H^+^ symporter |
| [*gfcB*](https://www.biocyc.org/ECOLI/substring-search?type=GENE&object=gfcB&geneSearch=Gene) | -1.711 | -1.225 | -1.196 |  |  | lipoprotein |
| [*ggt*](https://www.biocyc.org/ECOLI/substring-search?type=GENE&object=ggt&geneSearch=Gene) | 1.095 | 1.375 | -1.036 |  |  | periplasmic glutathione hydrolase proenzyme |
| [*grxD*](https://www.biocyc.org/ECOLI/substring-search?type=GENE&object=grxD&geneSearch=Gene) | 2.123 | 1.378 | 1.441 |  | X | glutaredoxin 4 |
| [*ilvB*](https://www.biocyc.org/ECOLI/substring-search?type=GENE&object=ilvB&geneSearch=Gene) | -2.32 | -2.162 | -2.403 | X |  | acetohydroxy acid synthase I subunit |
| [*ilvN*](https://www.biocyc.org/ECOLI/substring-search?type=GENE&object=ilvN&geneSearch=Gene) | -3.615 | -1.553 | -3.345 |  |  | acetohydroxy acid synthase I subunit |
| [*mprA*](https://www.biocyc.org/ECOLI/substring-search?type=GENE&object=mprA&geneSearch=Gene) | 1.898 | 1.235 | 1.205 |  |  | transcriptional repressor |
| [*ompA*](https://www.biocyc.org/ECOLI/substring-search?type=GENE&object=ompA&geneSearch=Gene) | 1.538 | 1.021 | 1.223 |  | X | outer membrane protein |
| [*ompC*](https://www.biocyc.org/ECOLI/substring-search?type=GENE&object=ompC&geneSearch=Gene) | 2.074 | 1.236 | 1.231 |  | X | outer membrane protein |
| [*pgaB*](https://www.biocyc.org/ECOLI/substring-search?type=GENE&object=pgaB&geneSearch=Gene) | -1.345 | 1.165 | -2.048 |  |  | lipoprotein/PGA hydrolysis |
| [*rluE*](https://www.biocyc.org/ECOLI/substring-search?type=GENE&object=rluE&geneSearch=Gene) | 1.15 | 1.148 | 1.023 |  |  | pseudouridine synthase |
| [*sdiA*](https://www.biocyc.org/ECOLI/substring-search?type=GENE&object=sdiA&geneSearch=Gene) | -1.76 | -1.038 | -1.307 |  |  | transcriptional regulator |
| [*tpiA*](https://www.biocyc.org/ECOLI/substring-search?type=GENE&object=tpiA&geneSearch=Gene) | 2.009 | 1.3 | 1.132 | X |  | triose-P isomerase |
| [*ugpA*](https://www.biocyc.org/ECOLI/substring-search?type=GENE&object=ugpA&geneSearch=Gene) | 9.027 | 8.631 | 1.344 |  |  | glycerol 3-P transporter |
| [*ugpC*](https://www.biocyc.org/ECOLI/substring-search?type=GENE&object=ugpC&geneSearch=Gene) | 7.627 | 7.103 | 1.323 |  |  | glycerol 3-P transporter |
| [*ugpE*](https://www.biocyc.org/ECOLI/substring-search?type=GENE&object=ugpE&geneSearch=Gene) | 8.476 | 8.765 | 1.428 |  |  | glycerol 3-P transporter |
| [*ugpQ*](https://www.biocyc.org/ECOLI/substring-search?type=GENE&object=ugpQ&geneSearch=Gene) | 6.045 | 5.628 | 1.33 |  |  | glycerol 3-P transporter |
| [*ybeQ*](https://www.biocyc.org/ECOLI/substring-search?type=GENE&object=ybeQ&geneSearch=Gene) | -2.324 | -1.44 | -1.438 |  |  | unknown |
| [*ybgD*](https://www.biocyc.org/ECOLI/substring-search?type=GENE&object=ybgD&geneSearch=Gene) | -3.568 | -3.623 | -2.9 |  |  | putative fimbrial gene |
| [*ybjC*](https://www.biocyc.org/ECOLI/substring-search?type=GENE&object=ybjC&geneSearch=Gene) | 2.063 | 1.046 | 1 | X |  | unknown |
| [*ycdT*](https://www.biocyc.org/ECOLI/substring-search?type=GENE&object=ycdT&geneSearch=Gene) | -2.581 | -1.268 | -2.072 |  |  | motility regulation |
| [*ydbD*](https://www.biocyc.org/ECOLI/substring-search?type=GENE&object=ydbD&geneSearch=Gene) | -2.526 | -1.053 | -2.428 |  |  | unknown |
| [*yfaH*](https://www.biocyc.org/ECOLI/substring-search?type=GENE&object=yfaH&geneSearch=Gene) | 5.649 | 6.654 | 1.316 |  |  | unknown |
| [*yfcL*](https://www.biocyc.org/ECOLI/substring-search?type=GENE&object=yfcL&geneSearch=Gene) | 1.213 | 1.122 | 1.171 |  |  | unknown |
| [*ygdG*](https://www.biocyc.org/ECOLI/substring-search?type=GENE&object=ygdG&geneSearch=Gene) | -1.528 | -1.069 | -1.078 |  |  | DNA endonuclease |
| [*yiaA*](https://www.biocyc.org/ECOLI/substring-search?type=GENE&object=yiaA&geneSearch=Gene) | -1.886 | -1.733 | -1.324 |  |  | inner membrane protein |
| [*yoeA*](https://www.biocyc.org/ECOLI/substring-search?type=GENE&object=yoeA&geneSearch=Gene) | 2.141 | 2.263 | 1.198 |  |  | pseudogene |

**Supplemental Table S9**. Strains used in this study

| Name | Genotype | Reference |
| --- | --- | --- |
| BL21 | *F^‐^ ompT lon gal dcm hsdS_B_(r_B_^‐^ m_B_^‐^) λ*(DE3 [*lacI lacUV5‐T7 gene 1 ind1 sam7 nin5*]) | Laboratory stock |
| BTH101 | *F^-^ cya-99 araD139 galE15 galK16 rpsL1 (Str^R^) hsdR2 mcrA1 mcrB1* | (Karimova et al. 1998) |
| BW25113 | *Δ(araD-araB)*567*, ΔlacZ*4787*(::rrnB*-3*), λ^−^, rph-1, Δ(rhaD-rhaB)*568*, hsdR*514 | (Baba et al. 2006) |
| JW0585 | BW25113 *ΔentC-731::kan* | (Baba et al. 2006) |
| JW2142 | BW25113 *ΔcirA-782::kan* | (Baba et al. 2006) |
| JW3379 | BW25113 *ΔmalQ-750::kan* | (Baba et al. 2006) |
| JW5134 | BW25113 *ΔcspH-732::kan* | (Baba et al. 2006) |
| R1279 | CSH50 *Δ*(*pho-bgl*)201 *Δ*(*lac-pro*) *ara thi* | (Schnetz et al. 1996) |
| TM338 | W3110 *mlc* *rne-FLAG-cat* | (Morita et al. 2004) |
| TM529 | W3110 *mlc* *rne598-FLAG-cat* | (Morita et al. 2004) |
| Xl1-blue | *recA1*, *endA1*, *gyrA96*, *thi-1*, *hsdR17*, *relA1*, *supE44*, *lac*, *F’*[*proAB lacI^q^ lacZΔM15 Tn10*] | Laboratory stock |
| Z8 | R1279 *λattB::*[*aadA, glmS’-lacZ*], *strp^R^, F'*(*lacI^q^*) | (Kalamorz et al. 2007) |
| Z28 | R1279 *ΔrapZ λattB::*[*aadA, glmS*’-*lacZ*], *strp^R^ , F'*(*lacI^q^*) | (Kalamorz et al. 2007) |
| Z37 | R1279 *ΔrapZ* | (Kalamorz et al. 2007) |
| Z64 | R1279 *rne-FLAG-cat* | (Göpel et al. 2013) |
| Z106 | R1279 *ΔglmY ΔglmZ* | (Göpel et al. 2013) |
| Z864 | R1279 *ΔglmY ΔglmZ ΔrapZ* | (Göpel et al. 2016) |
| Z903 | R1279 *ΔrapZ rne598-FLAG-cat* | (Gonzalez et al. 2017) |
| Z1241 | R1279 *rne598-FLAG-cat* | T4GT7(TM529)🡪R1279; this work |
| Z1242 | R1279 *ΔrapZ* *rne-FLAG-cat* | T4TG7(TM338)🡪Z37; this work |

**Supplemental Table S10**. Plasmids used in this study

| Name | Relevant structure^a^ | | Reference |
| --- | --- | --- | --- |
| pBAD33 | *P_Ara_*, MCS 2, *cat*, ori p15A | (Guzman et al. 1995) | |
| pBGG61 | *rapZ* under *P_Ara_* control in pBAD33 | (Göpel et al. 2013) | |
| pBGG164 | *strep-rapZ* under *P_tac_* control, *lacI^q^*, *bla*, ori ColEI | (Lüttmann et al. 2012) | |
| pBGG190 | *His_10_-ptsN* under *P_tac_* control, *lacI^q^*, *bla*, ori ColEI | (Lüttmann et al. 2009) | |
| pBGG217 | *strep-ptsN* under *P_tac_* control, *lacI^q^*, *bla*, ori ColEI | (Lüttmann et al. 2012) | |
| pBGG237 | *strep-tag* under *P_tac_* control, *lacI^q^*, *bla*, *ori* ColEI | (Lüttmann et al. 2012) | |
| pBGG348 | encodes T25-RapZ in pKT25 | (Göpel et al. 2013) | |
| pBGG349 | encodes T18-RapZ in pUT18C | (Göpel et al. 2013) | |
| pKT25 | *P_lac_*::*cyaA* [*1-224*] (T25), MCS, *neo*, *ori* p15A | (Karimova et al. 1998) | |
| pKT25-zip | encodes T25-GCN4 leucine zipper fusion in pKT25 | (Karimova et al. 1998) | |
| pRne529-N | *His_6_-rne_NTD_* (aa 1-529) in pET16b, *bla*, ori ColEI | (Callaghan et al. 2003) | |
| pSD2 | encodes T25-RNase E (aa 1-762) fusion in pKT25 | this work | |
| pSD3 | encodes T25-RNase E (aa 1-415) fusion in pKT25 | this work | |
| pSD5 | encodes T25-RNase E (aa 1-400) fusion in pKT25 | this work | |
| pSD6 | encodes T25-RNase E (aa 1-279) fusion in pKT25 | this work | |
| pSD7 | encodes T25-RNase E (aa 118-597) fusion in pKT25 | this work | |
| pSD8 | encodes T25-RNase E (aa 215-597) fusion in pKT25 | this work | |
| pSD9 | encodes T25-RapZ (aa 1-152) fusion in pKT25 | (Gonzalez et al. 2017) | |
| pSD10 | encodes T25-RapZ (aa 153-284) fusion in pKT25 | (Gonzalez et al. 2017) | |
| pSD11 | encodes T18-RapZ (aa 1-152) fusion in pUT18C | (Gonzalez et al. 2017) | |
| pSD12 | encodes T18-RapZ (aa 153-284) fusion in pUT18C | (Gonzalez et al. 2017) | |
| pSD18 | encodes T25-RNase E (aa 1-597) R169Q fusion in pKT25 | this work | |
| pSD19 | encodes T25-RNase E (aa 1-597) T170V fusion in pKT25 | this work | |
| pSD23 | *His_10_-rne_NTD_* (aa 1-529) under *P_tac_* control, *lacI^q^*, *bla*, ori ColEI | (Durica-Mitic and Görke 2019) | |
| pSD24 | *strep-rapZ_CTD_* under *P_tac_* control, *lacI^q^*, *bla*, ori ColEI | (Gonzalez et al. 2017) | |
| pSD25 | *strep-rapZ_NTD_* under *P_tac_* control, *lacI^q^*, *bla*, ori ColEI | (Gonzalez et al. 2017) | |
| pSD26 | *rapZ_NTD_* under *P_Ara_* control in pBAD33 | this work | |
| pSD27 | *rapZ_CTD_* under *P_Ara_* control in pBAD33 | this work | |
| pSD39 | as pSD10 but RapZ_CTD_ with Phe169Ser substitution | this work | |
| pSD41 | as pSD10 but RapZ_CTD_ with Leu186Ser substitution | this work | |
| pSD43 | as pSD10 but RapZ_CTD_ with Met198Thr substitution | this work | |
| pSD44 | as pSD10 but RapZ_CTD_ with Gly200Asp substitution | this work | |
| pSD45 | as pSD10 but RapZ_CTD_ with Trp229Arg substitution | this work | |
| pSD71 | as pSD12 but RapZ_CTD_ with Leu186Ser substitution | this work | |
| pSD73 | as pSD12 but RapZ_CTD_ with Phe169Ser substitution | this work | |
| pSD74 | as pSD12 but RapZ_CTD_ with Val163Asp substitution | this work | |
| pSD75 | as pSD10 but RapZ_CTD_ with Val163Asp substitution | this work | |
| pSD76 | as pSD12 but RapZ_CTD_ with Met198Thr substitution | this work | |
| pSD77 | as pSD12 but RapZ_CTD_ with Gly200Asp substitution | this work | |
| pSD78 | as pSD12 but RapZ_CTD_ with Trp229Arg substitution | this work | |
| pSD101 | *strep-rapZ_CTD_* with quadruple mutation under *P_tac_* control, *lacI^q^*, *bla*, ori ColEI | this work | |
| pSD111 | encodes T18-RapZ (aa 6-284) fusion in pUT18C | this work | |
| pSD112 | encodes T18-RapZ (aa 11-284) fusion in pUT18C | this work | |
| pSD113 | encodes T18-RapZ (aa 1-279) fusion in pUT18C | this work | |
| pSD114 | encodes T18-RapZ (aa 1-274) fusion in pUT18C | this work | |
| pSD116 | encodes T25-RapZ (aa 6-284) fusion in pKT25 | this work | |
| pSD117 | encodes T25-RapZ (aa 11-284) fusion in pKT25 | this work | |
| pSD118 | encodes T25-RapZ (aa 1-279) fusion in pKT25 | this work | |
| pSD119 | encodes T25-RapZ (aa 1-274) fusion in pKT25 | this work | |
| pSD128 | *rapZ_1-279_* under *P_Ara_* control in pBAD33 | this work | |
| pSD132 | encodes T25-RapZ (aa 1-276) fusion in pKT25 | this work | |
| pSD133 | encodes T18-RapZ (aa 1-276) fusion in pUT18C | this work | |
| pSD135 | *strep-rapZ_1-279_* under *P_tac_* control, *lacI^q^*, *bla*, ori ColEI | this work | |
| pSD140 | encodes T18-RapZ (aa 1-278) fusion in pUT18C | this work | |
| pSD141 | encodes T18-RapZ (aa 1-277) fusion in pUT18C | this work | |
| pSD142 | encodes T25-RapZ (aa 1-278) fusion in pKT25 | this work | |
| pSD143 | encodes T25-RapZ (aa 1-277) fusion in pKT25 | this work | |
| pSD153 | *rapZ_1-278_* under *P_Ara_* control in pBAD33 | this work | |
| pSD154 | *rapZ_1-277_* under *P_Ara_* control in pBAD33 | this work | |
| pSD157 | *strep-rapZ_1-278_* under *P_tac_* control, *lacI^q^*, *bla*, ori ColEI | this work | |
| pSD158 | *strep-rapZ_1-277_* under *P_tac_* control, *lacI^q^*, *bla*, ori ColEI | this work | |
| pSD184 | *strep-rne_NTD_* (aa 1-529)-T170V under *P_tac_* control, *lacI^q^*, *bla*, ori ColEI | this work | |
| pSD191 | encodes T25-RNase E (aa 416-1061) fusion in pKT25 | this work | |
| pSD192 | encodes T18-RapZ (aa 1-279) L279G in pUT18C | this work | |
| pSD193 | encodes T18-RapZ (aa 1-279) T278G in pUT18C | this work | |
| pSD194 | encodes T18-RapZ (aa 1-279) T278G L279G in pUT18C | this work | |
| pSD195 | *strep-rapZ* (aa 153-279) under *P_tac_* control, *lacI^q^*, *bla*, ori ColEI | this work | |
| pSD196 | *strep-rne_NTD_* (aa 1-529)-R169Q under *P_tac_* control, *lacI^q^*, *bla*, ori ColEI | this work | |
| pSD197 | encodes T25-RapZ (aa 1-279) L279G in pKT25 | this work | |
| pSD198 | encodes T25-RapZ (aa 1-279) T278G in pKT25 | this work | |
| pSD199 | encodes T25-RapZ (aa 1-279) T278G L279G in pKT25 | this work | |
| pSD205 | *His_10_-rne_NTD_* (aa 1-529)-R169Q under *P_tac_* control, *lacI^q^*, *bla*, ori ColEI | this work | |
| pSD206 | *His_10_-rne_NTD_* (aa 1-529)-T170V under *P_tac_* control, *lacI^q^*, *bla*, ori ColEI | this work | |
| pUT18C | *P_lac_*::*cyaA* [*225-399*] (T18), MCS, *bla*, *ori* ColEI | (Karimova et al. 1998) | |
| pUT18-zip | encodes T18-GCN4 leucine zipper fusion in pUT18C | (Karimova et al. 1998) | |
| pYG29 | *strep-rapZ_quad_* under *P_tac_* control, *lacI^q^*, *bla*, ori ColEI | (Göpel et al. 2013) | |
| pYG30 | *rapZ_quad_* under *P_Ara_* control in pBAD33 | (Göpel et al. 2013) | |
| pYG39 | encodes T18-RapZ_quad_ fusion in pUT18C | this work | |
| pYG57 | *rapZ*-Ser7Asn under *P_Ara_* control in pBAD33 | this work | |
| pYG94 | encodes T25-RapZ_quad_ fusion in pKT25 | (Göpel et al. 2013) | |
| pYG97 | encodes T18-RNase E (aa 1-597) fusion in pUT18C | (Göpel et al. 2013) | |
| pYG98 | encodes T18-RNase E (aa 499-1061) fusion in pKT25 | (Göpel et al. 2013) | |
| pYG99 | encodes T18-RNase E (full-length) fusion in pUT18C | (Göpel et al. 2013) | |
| pYG100 | encodes T25-RNase E (full-length) fusion in pKT25 | this work | |
| pYG101 | encodes T25-RNase E (aa 1-597) fusion in pKT25 | this work | |
| pYG102 | encodes T25-RNase E (aa 499-1061) fusion in pKT25 | this work | |
| pYG189 | *strep-rng* under *P_tac_* control, *lacI^q^*, *bla*, ori ColEI | this work | |
| pYG201 | encodes T18-RNase E (aa 1-597) K112A fusion in pUT18C | this work | |
| pYG202 | encodes T25-RNase E (aa 1-597) K112A fusion in pKT25 | this work | |
| RapZ 1.3_pBAD33 | *rapZ-*Met198Thr under *P_Ara_* control in pBAD33 | this work | |
| RapZ 1.5_pBAD33 | *rapZ-*Leu123Gln under *P_Ara_* control in pBAD33 | this work | |
| RapZ 1.8_pBAD33 | *rapZ-*Leu241Ser under *P_Ara_* control in pBAD33 | this work | |
| RapZ 2.4_pBAD33 | *rapZ-*Phe169Ser under *P_Ara_* control in pBAD33 | this work | |
| RapZ 2.5_pBAD33 | *rapZ-*Leu186Ser under *P_Ara_* control in pBAD33 | this work | |
| RapZ 2.6_pBAD33 | *rapZ-*Gly200Asp under *P_Ara_* control in pBAD33 | this work | |
| RapZ 2.10_pBAD33 | *rapZ-*Trp229Arg under *P_Ara_* control in pBAD33 | this work | |
| RapZ 4.3_pBAD33 | *rapZ-*Leu102Pro under *P_Ara_* control in pBAD33 | this work | |
| RapZ 5.6_pBAD33 | *rapZ-*Val163Asp under *P_Ara_* control in pBAD33 | this work | |

^a^*ori*: origin of replication; *RBS*: ribosomal binding site, MCS: multiple cloning site

**Supplemental Table S11**. Oligonucleotides used in this study

| Primer | Sequence^a^ | Res. Sites | Position^b^ |
| --- | --- | --- | --- |
| BG149 | CTGGCGCGGAAGTAAAACG |  | *glmS* 676 to 694 |
| BG150 | CTAATACGACTCACTATAGGGAGAAGAACCCGGAACGTTA |  | *glmS*1144 to 1125 |
| BG168 | GCCTCGAGATGGTACTGATGATCGTCAGC | XhoI | *rapZ* 1 to 21 |
| BG230 | GTAGATGCTCATTCCATCTC |  | *glmZ* 1 to 20 |
| BG231 | CTAATACGACTCACTATAGGGagAAAACAGGTCTGTATGACAAC |  | *glmZ* 172 to 152 |
| BG260 | AGTGGCTCATTCACCGAC |  | *glmY* 1 to 18 |
| BG261 | CTAATACGACTCACTATAGGGAGATAAGGCGGTGCCTAACTC |  | *glmY* 150 to 131 |
| BG287 | TGCCTGGCGGCCGTAG |  | *rrfD* 1 to 16 |
| BG288 | CTAATACGACTCACTATAGGGAGAGCCTGGCAGTTCCCTAC |  | *rrfD* 118 to 102 |
| BG397 | TGGCTGCAGTCTAGATTATCATGGTTTACGTTTTTCCAGCG | PstI, XbaI | *rapZ* 855 to 833 |
| BG444 | CTAATACGACTCACTATAGGGAGAGTAGATGCTCATTCCATCTCTTATG |  | *glmZ* 1 to 25 |
| BG445 | AAAAAAACGCCTGCTCTTATTACGGAGC |  | *glmZ* 207 to 180 |
| BG637 | GCGTCTAGAGATGGTACTGATGATCGTCAGCG | XbaI | *rapZ* 1 to 22 |
| BG639 | CGCGGTACCTCATGGTTTACGTTTTTCCAGCG | KpnI | *rapZ* 855 to 835 |
| BG646 | TTCCGCGACTCGGCGCGC |  | pKT25 927-944 |
| BG647 | GGGGATGTGCTGCAAGGCG |  | pKT25 1124-1106 |
| BG861 | CGCCTGCAGATGGTACTGATGATCGTCAGC | PstI | *rapZ* 1 to 21 |
| BG920 | GGCTCTAGATTATCATGGTGCAGCTGCTTCCAGCGTACGATGGCGTGACTGGACGTTTGCACCGCGCGAGCGGAAGTAG | XbaI | *rapZ* 855 to 789 |
| BG1049 | GGCGAGCTCGTGAGGAGAAACAGTACATGGTACTGATGATCGTCAGCG | SacI | *rapZ* -17 to +22 |
| BG1056 | GGCGAGCTCGTGAGGAGAAACAGTACATGGTACTGATGATCGTCA | SacI | *rapZ* -17 to +19 |
| BG1110 | GCGTCTAGAGATGAAAAGAATGTTAATCAACGC | XbaI | *rne* 1 to 23 |
| BG1111 | cgcGGTACCTTACTCAACAGGTTGCGGAC | KpnI | *rne* 3186 to 3167 |
| BG1114 | CGCGGTACCTTATTTCGGTGCTGGTTGCTCGG | KpnI | *rne* 1791 to 1774 |
| BG1142 | CGCGGTACCTTAGCGTGGAGCTGGCGCTTC | KpnI | *rne* 2286 to 2269 |
| BG1143 | CGCGGTACCTTAGTCACGCACGGTGCCAG | KpnI | *rne* 1245 to 1229 |
| BG1159 | CGCGGTACCTTAACTGGATTCACCCAGTGATGG | KpnI | *rne* 1200 to 1180 |
| BG1160 | CGCGGTACCTTAGAAGGCGGACTCGATCTGTG | KpnI | *rne* 837 to 818 |
| BG1161 | GGCTGCTAGCATGAAAAGAATGTTAATCAACGC | NheI | *rne* 1 to 23 |
| BG1173 | GCGTCTAGAGACCTTTATCAGTCTGGCGGG | XbaI | *rne* 352 to 371 |
| BG1174 | GCGTCTAGAGGTTCGCGCATTCCGCGATTAC | XbaI | *rne* 644 to 663 |
| BG1223 | GCGTCTAGAGCTGGGTAAACGTGAACGCG | XbaI | *rapZ* 457 to 475 |
| BG1250 | GGCGAATTCAAAAAAACCCGCTTGCGCGGG | EcoRI | *rpsT* 315 to 295 |
| BG1287 | GGCTGCTAGCATGACGGCTGAATTGTTAGTAAAC | NheI | *rng* 1 to 24 |
| BG1288 | CCAAGCTTTCTAGATTACATCATTACGACGTCAAACTG | HindIII, XbaI | *rng* 1470 to 1447 |
| BG1299 | [P]-CATGGGGCTTATCGTGC**AA**ACCGCTGGCGTCGGC |  | *rne* 489 to 522 |
| BG1300 | [P]-CATGGGGCTTATCGTGCGC**GT**CGCTGGCGTCGGCAAATC |  | *rne* 489 to 527 |
| BG1330 | [P]-GAAGAGCGCGGCAAC**GC**AGGCGCGGCATTAAC |  | *rne* 319 to 350 |
| BG1340 | GGCTGCTAGCCTGGGTAAACGTGAACGCGAAC | NheI | *rapZ* 457 to 478 |
| BG1358 | GCGTCTAGAttacagacgggtacgcagcattt | XbaI | *rapZ* 456 to 437 |
| BG1359 | GGCGAGCTCGTGAGGAGAAACAGTACATGCTGGGTAAACGTGAACGCGAAC | SacI | *rapZ* -17 to -1 and +457 to +478 |
| BG1511 | ATCCTAATACGACTCACTATAGGGGCGGGAATAGCTCAGTTGG |  | *glyX* 1 to 19 |
| BG1512 | GCGTCGCTGTGGATATTTTATT |  | *glyY* 110 to 89 |
| BG1585 | GCGTCTAGAGGTCAGCGGACGTTCAGGTTC | XbaI | *rapZ* 16 to 35 |
| BG1586 | GCGTCTAGAGGGTTCAGGTAAATCTGTCGCC | XbaI | *rapZ* 31 to 51 |
| BG1587 | CGCGGTACCTTACAGCGTACGATGGCGTGAC | KpnI | *rapZ* 837 to 819 |
| BG1588 | CGCGGTACCTTATGACTGGACGTTTTTACCGCG | KpnI | *rapZ* 822 to 802 |
| BG1618 | TGGCTGCAGTCTAGATTACAGCGTACGATGGCGTGAC | PstI, XbaI | *rapZ* 837 to 819 |
| BG1619 | CGCGGTACCTTAATGGCGTGACTGGACGTTTTTAC | KpnI | *rapZ* 828 to 806 |
| BG1633 | GGCTCTAGAGGTACCTTACGTACGATGGCGTGACTGG | KpnI, XbaI | *rapZ* 834 to 816 |
| BG1634 | GGCTCTAGAGGTACCTTAACGATGGCGTGACTGGACG | KpnI, XbaI | *rapZ* 831 to 813 |
| BG1684 | ATCCTAATACGACTCACTATAGGGGGATACTAAAACAGGAGGTTTTATG |  | *uspF* -22 to +3 |
| BG1685 | AAAGCCCGCAGCAATGTGCGG |  | *uspF* 465 to 445 |
| BG1686 | CTAATACGACTCACTATAGGGagAGCCATCACTACGTAACGAG |  | *rpsT -*132 to -113 |
| BG1901 | GGCCTGCAGTCTAGA**TTA**CAGCGCAGGTTGTTCCGGA | PstI, XbaI | *rne* 1587 to 1569 |
| BG1904 | CGCGGTACCTTAGCCCGTACGATGGCGTGACTGGA |  | *rapZ* 834 to 815 |
| BG1905 | CGCGGTACCTTACAGGCCACGATGGCGTGACTGGACGT |  | *rapZ* 837 to 812 |
| BG1906 | CGCGGTACCTTAGCCGCCACGATGGCGTGACTGGACGT |  | *rapZ* 831 to 812 |
| BG1908 | GTTGCTCCTTCCTGTGCCAG |  | *fimA* 212 to 193 |
| BG1909 | CGTTAGCTTTACATATAGCGGAG |  | *fimA* -274 to -252 |
| BG1910 | AGTTTGTCCGCGATGCTTTCC |  | *fimA* -163 to -143 |
| BG1911 | CGAACTGAAATACTACGGTGAG |  | *cirA* 870 to 891 |
| BG1912 | CTAATACGACTCACTATAGGGAGATGATCGTCCATACGCACGCC |  | *cirA* 1160 to 1141 |
| BG1915 | AGTACAGCAGACCATGGCAAC |  | *entC* 24 to 44 |
| BG1916 | CTAATACGACTCACTATAGGGAGAGTTCCACCACATTCAGCGAC |  | *entC* 358 to 339 |
| BG1917 | TGACGCGATGCATCAACGTAC |  | *malQ* 114 to 134 |
| BG1918 | CTAATACGACTCACTATAGGGAGACGATCGCAGCGTATAAAGCTG |  | *malQ* 465 to 445 |
| BG1919 | CCTCAGTTCTACAGCGGCTC |  | *fimA* 45 to 64 |
| BG1920 | CTAATACGACTCACTATAGGGAGACCAGGATCTGCACACCAACG |  | *fimA* 382 to 363 |
| BG1927 | AGTGCAGCGCAGTATTTTCAGT |  | *cspH* -34 to -12 |
| BG1928 | CTAATACGACTCACTATAGGGAGACATTTGCCGCTGTTGGTCCTC |  | *cspH* 199 to 179 |
| BG1931 | GCGTCTAGAGAACGAATCGCTGTCGCTCTC |  | *rne* 1246 to 1265 |
| BG1942 | GCCGAAAGGCTCAAGTTAAGG |  | *cspB* -29 to -9 |
| BG1943 | CTAATACGACTCACTATAGGGAGAGCACCACTCTCTATAGAGAAGG |  | *cspB* 176 to 155 |

^a^Restriction sites are underlined. The recognition site for T7 RNA polymerase is underlined by a dashed line. ^b^Positions are relative to the first nucleotide of the respective gene as annotated in the EcoCyc database (Keseler et al. 2017). [P] indicates 5’-phosphorylation of the oligonucleotide. Positions deviating from the *wild type* coding sequence are shown in bold.

**SUPPLEMENTAL MATERIALS AND METHODS**

***Construction of plasmids***

*E. coli* XL1 blue was used for cloning recombinant plasmids. BACTH plasmids were constructed by insertion of the DNA fragment encoding the candidate protein of choice between the XbaI/KpnI sites on plasmid pKT25 or pUT18C, thereby generating in-frame fusions with the upstream encoded T25- and T18-fragments, respectively. As an exception, plasmid pYG39 was obtained by amplification of *rapZ_quad_* using primers BG861/BG920 and subsequent ligation of the PCR fragment between the PstI/XbaI sites on plasmid pUT18C. The following primers were used for amplification of truncated *rapZ* alleles: BG1585/BG639 (RapZ_6-284_), BG1586/BG639 (RapZ_11-284_), BG637/BG1587 (RapZ_1-279_), BG637/BG1633 (RapZ_1-278_), BG637/BG1634 (RapZ_1-277_), BG637/BG1619 (RapZ_1-276)_ and BG637/BG1588 (RapZ_1-274_). The substitutions of residues 278 and 279 within RapZ_1-279_ were introduced via the reverse primer using primer combinations BG637/BG1904 for L279G, BG637/BG1905 for T278G and BG637/BG1906 for the double substitution. The PCR fragments were cloned between the XbaI/KpnI sites on plasmid pUT18C or pKT25. Subsequently, the XbaI-KpnI fragments of the latter plasmids were isolated and inserted between the XbaI/KpnI sites of the complementary BACTH plasmid. These constructions resulted in the following pUT18C derivatives: pSD111 (T18-RapZ_6-284_), pSD112 (T18-RapZ_11-284_), pSD113 (T18-RapZ_1-279_), pSD140 (T18-RapZ_1-278_), pSD141 (T18-RapZ_1-277_), pSD133 (T25-RapZ_1-276_), pSD114 (T18-RapZ_1-274_), pSD192 (T18-RapZ_1-279_ L279G), pSD193 (T18-RapZ_1-279_ T278G) and pSD194 (T18-RapZ_1-279_ T278G L279G). Moreover, the following pKT25 derivatives encoding the same *rapZ* truncations, but fused to T25, were obtained: pSD116, pSD117, pSD118, pSD142, pSD143, pSD132, pSD119, pSD197, pSD198 and pSD199. For construction of T25-RapZ_CTD_ variants carrying mutations identified in the *rapZ* random mutagenesis screen (Supplemental Table S1), the respective alleles were amplified from isolates 2.2, 2.5, 1.3, 2.6, 2.10 and 5.6 using primers BG1223/BG639 and cloned between the XbaI/Kpn sites of pKT25, respectively. Resulting plasmids were pSD39, pSD41, pSD43, pSD44, pSD45 and pSD75. The XbaI-KpnI fragments of the latter plasmids were subsequently isolated and ligated with XbaI/KpnI digested pUT18C, resulting in plasmids pSD73, pSD71, pSD76, pSD77, pSD78 and pSD74, respectively.

The BACTH plasmids encoding truncated RNase E variants fused to T25 were obtained by insertion of PCR fragments generated with the following primers between the XbaI/KpnI sites on plasmid pKT25, resulting in the plasmids mentioned in parentheses: BG1110/BG1142 (pSD2; T25-Rne_1-729_), BG1110/BG1143 (pSD3; T25-Rne_1-415_), BG1110/BG1159 (pSD5; T25-Rne_1-400_), BG1110/BG1160 (pSD6; T25-Rne_1-279_), BG1173/BG1114 (pSD7; T25-Rne_118-597_), BG1174/BG1114 (pSD8; T25-Rne_215-597_) and BG1931/BG1111 (pSD191; T25-Rne_416-1061_). Plasmids pYG100 (T25-Rne_FL_), pYG101 (T25-Rne_1-597_), pYG102 (T25-Rne_499-1061_) were obtained by ligating the XbaI-KpnI inserts of plasmids pYG99, pYG97 and pYG98 with XbaI/KpnI-digested pKT25, respectively. The K112A, R169Q and T170V substitutions were introduced into *rne_NTD_* by site-directed mutagenesis using the combined chain reaction approach (Bi and Stambrook 1998). Briefly, the mutations were introduced by incorporation of the 5’-phosphorylated mutagenesis primer BG1330, BG1299 or BG1300 by thermostable ampligase (Epicentre) during PCR with the external primers BG1110/BG1114. The resulting DNA fragments were cloned between the XbaI/KpnI sites of plasmid pUT18C or pKT25, generating plasmids pYG201, pYG202, pSD18 and pSD19.

For construction of pBAD33 derivatives directing expression of *rapZ* variants from the *P_Ara_* promoter, the respective *rapZ* alleles were amplified using primers BG1049/BG1358 (*rapZ_NTD_*), BG1359/BG397 (*rapZ_CTD_*), BG1049/BG1618 (*rapZ_1-279_*), BG1049/BG1633 (*rapZ_1-278_*), and BG1049/BG1634 (*rapZ_1-277_*). Subsequently, the PCR fragments were inserted between the SacI/XbaI sites on pBAD33 resulting in plasmids pSD26, pSD27, pSD128, pSD153 and pSD154, respectively. The BamHI-XbaI fragments of plasmids pSD128, pSD153 and pSD154 comprising the 3’ ends of the respective *rapZ* alleles were isolated and used to replace the BamHI-XbaI fragment of plasmid pBGG164, resulting in plasmids pSD135, pSD157 and pSD158, respectively. The pBAD33 derivatives triggering expression of the *rapZ* mutants identified in the random mutagenesis screen were obtained by PCR amplification of the isolates 4.3, 1.5, 5.6, 2.4, 2.5, 1.3, 2.6, 2.10 and 1.8 (Supplemental Table S1) using primers BG1049/BG397 and cloning of the obtained DNA fragments between the SacI/XbaI sites on pBAD33, respectively. The resulting plasmids were named according to the isolate name (e.g. RapZ 1.3_pBAD33; Supplemental Table S10). The *rapZ-S7N* mutant was amplified using primers BG1056/BG397 and cloned between the SacI/XbaI sites of plasmid pBAD33 resulting in plasmid pYG57. Plasmids pSD101 and pSD195 encode Strep-RapZ_CTDquad_ and Strep-RapZ_CTD153-279_ under *P_tac_* control, respectively. They were generated by inserting the PCR fragments obtained with primers BG1340/BG920 and BG1340/BG1618 (template: pSD24), respectively, between the NheI/XbaI sites on plasmid pBGG237. For construction of plasmid pYG189, the *rng* gene was amplified using primers BG1287/BG1288 and the DNA fragment was subsequently ligated with NheI and HindIII digested plasmid pBGG237. Plasmids pSD205 and pSD206, which trigger overproduction of N-terminally His_10_-tagged Rne_1-529_ variants carrying the R169Q and T170V substitutions, were constructed in two steps. First, the respective *rne* alleles were amplified by PCR using primers BG1161/BG1901 and plasmids pSD18 and pSD19 as templates, respectively. The PCR fragments were cloned between the NheI/PstI sites on pBGG237 resulting in plasmids pSD196 and pSD184, which encode Strep-tagged versions of the *rne_1-529_* variants. Finally, the NheI-PstI fragments comprising the *rne_1-52_*_9_ sequence were isolated from the latter plasmids and used to replace the corresponding fragment in pBGG190.

***Purification of Strep-tagged proteins***

Strep-tagged proteins were overproduced in strains Z106 or Z864 using the following plasmids: pBGG164 (RapZ), pYG29 (RapZ_quad_), pSD135 (RapZ_1-279_), pBGG217 (PtsN), pYG189 (RNase G), pSD24 (Strep-RapZ_CTD_), pSD25 (Strep-RapZ_NTD_), pSD101 (Strep-RapZ_CTDquad_), pSD195 (Strep-RapZ_CTD 153-279_), pSD157 (RapZ_1-278_), pSD158 (RapZ_1-277_). Cultures were grown in 100-500 ml LB until OD_600_ = 0.5-0.8 and protein synthesis was induced by 1 mM IPTG. After 1 h cells were harvested by centrifugation (4000 rpm, 20 min, 4°C) and resuspended in buffer W (100 mM Tris-HCl pH 8.0, 150 mM NaCl, 1 mM EDTA). Following cell disruption by using a French pressure cell, a OneShot model cell disruptor or sonication, lysates were cleared by centrifugation steps (4000 rpm, 4°C, 20 min, Eppendorf 5810R and 14 000 rpm, 4°C, 1 h, Eppendorf 5427R). The supernatants were loaded on poly-prep chromatography columns (Bio-Rad) containing pre-equilibrated StrepTactin sepharose matrix (IBA) corresponding to 1/1000 of the original culture volume. The matrix was washed 4× using 10× column bed volumes (CBV) of buffer W and proteins were eluted in three steps using 1 CBV of buffer E (100 mM Tris-HCl pH 8.0, 150 mM NaCl, 1 mM EDTA, 2.5 mM desthiobiotin) each. Proteins were usually dialyzed 2× for 20 h at 4°C in dialysis buffer (10 mM Tris-HCl pH 7.0, 100 mM KCl, 10 mM MgCl_2_, 2 mM β-mercaptoethanol). Afterwards, proteins were mixed with glycerol (10% v/v), shock-frozen and stored at -80°C.

***Purification of His-tagged RNase E-NTD (aa 1-529)***

His-tagged RNase E (1-529) and its variants were overproduced in strain Z106 or BL21 using plasmids pRne529-N, pSD23, pSD205 and pSD206. Purification was performed as described previously (Göpel et al. 2016). Briefly, protein synthesis was induced when cultures reached OD_600_ = 0.5-0.8. Following an additional 1 h of growth, cells were harvested by centrifugation, resuspended in ZAP buffer (50 mM Tris-HCl pH 7.5, 200 mM NaCl) and disrupted using a French pressure cell or OneShot model cell disruptor. Lysates were cleared by centrifugation steps (4000 rpm, 4°C, 20 min, Eppendorf 5810R and 35000 rpm, 4°C, 1 h, Beckman 60TI) and loaded on poly-prep chromatography columns (Bio-Rad) containing 3 ml Ni-NTA superflow suspension (IBA) pre-equilibrated with 2× 10 ml ZAP buffer. Subsequently, columns were 5× washed with 10 ml ZAP buffer containing incremental concentrations of imidazole (5, 10, 25, 50 and 80 mM) and finally eluted using 3× 2.5 ml ZAP buffer containing 125, 250 and 500 mM imidazole, respectively. The 250 mM fraction containing the highest His_6_-Rne_NTD_ yield was dialyzed against 20 mM Tris-HCl pH 7.9, 500 mM NaCl, 10 mM MgCl_2_, 0.5 mM EDTA and 10 mM DTT or 20 mM β-mercaptoethanol (2×, 20 h, 4°C). Dialyzed protein was mixed with glycerol (5% v/v) and frozen at -80°C.

***Whole transcriptome analysis by RNA-sequencing***

Strains were grown in LB until cultures reached OD_600_ = 0.3. Subsequently, 1 mM IPTG was added to induce overproduction of plasmid-encoded RapZ variants in the respective transformants and cultures were harvested following additional 30 min of growth. Total RNA was isolated from two biological replicates each, using the ReliaPrep RNA Cell Miniprep System (Promega) according to the manufacturer´s instructions with minor alterations that included cell lysis in TE buffer containing 400 μg/ml lysozyme and omission of DNase I treatment. DNA was removed using Turbo DNA-*free* Kit (Invitrogen) according to manufacturer´s instructions. Library preparation and sequencing was performed at the Vienna Biocenter Campus Science Support Facility (https://www.viennabiocenter.org/facilities/next-generation-sequencing/). Ribo-Zero^®^ rRNA removal kit (Illumina) was used to deplete ribosomal RNA. cDNA libraries were constructed using the NEBNext^®^ Ultra^™^ II Directional RNA Library Prep Kit (Illumina). 100 bp single end sequence reads were generated using the Illumina HiSeqV4 SR100 platform. Read quality was assessed with FastQC (<https://www.bioinformatics.babraham.ac.uk/projects/fastqc/>). Adaptor sequences and poor quality reads were removed using trimmomatic (Bolger et al. 2014). Remaining reads were mapped to the *E. coli* reference genome (accession number NC_000913.3) using short read aligner Segemehl (Otto et al. 2014). Reads mapping to multiple locations in the genome equally well were removed. Read counts per annotated gene were deduced using HTSeq-count (Anders et al. 2015). TPM values (transcript per million) were calculated using the Vienna NGS package (Wolfinger et al. 2015). Differential gene expression analysis was performed with DESeq2 (Love et al. 2014). The levels of transcripts with a |log2 fold change| ≥ 1 and an adjusted *p*-value < 0.05 were considered to be significantly altered. The raw sequencing data were deposited to the European Nucleotide Archive (ENA; https:// www.ebi.ac.uk/ena) under the accession number PRJEB35302. The list of potential RNase E direct entry cleavage sites was obtained from Kenneth McDowall (Clarke et al. 2014) including coordinates corresponding to the *E. coli* reference genome NC_000913.2. To allocate these sites to annotated genes, the matching *E. coli* genome annotation (U00096.2) was retrieved and used together with bedtools closest (Quinlan and Hall 2010) using parameters -s -D b -t all.

**SUPPLEMENTAL REFERENCES**

Anders S, Pyl PT, Huber W. 2015. HTSeq--a Python framework to work with high-throughput sequencing data. *Bioinformatics* **31**: 166-169.

Baba T, Ara T, Hasegawa M, Takai Y, Okumura Y, Baba M, Datsenko KA, Tomita M, Wanner BL, Mori H. 2006. Construction of *Escherichia coli* K-12 in-frame, single-gene knockout mutants: the Keio collection. *Mol Syst Biol* **2**: 1-11.

Bi W, Stambrook PJ. 1998. Site-directed mutagenesis by combined chain reaction. *Anal Biochem* **256**: 137-140.

Bolger AM, Lohse M, Usadel B. 2014. Trimmomatic: a flexible trimmer for Illumina sequence data. *Bioinformatics* **30**: 2114-2120.

Callaghan AJ, Grossmann JG, Redko YU, Ilag LL, Moncrieffe MC, Symmons MF, Robinson CV, McDowall KJ, Luisi BF. 2003. Quaternary structure and catalytic activity of the *Escherichia coli* ribonuclease E amino-terminal catalytic domain. *Biochemistry* **42**: 13848-13855.

Clarke JE, Kime L, Romero AD, McDowall KJ. 2014. Direct entry by RNase E is a major pathway for the degradation and processing of RNA in *Escherichia coli*. *Nucleic Acids Res* **42**: 11733-11751.

Deana A, Celesnik H, Belasco JG. 2008. The bacterial enzyme RppH triggers messenger RNA degradation by 5' pyrophosphate removal. *Nature* **451**: 355-358.

Durica-Mitic S, Görke B. 2019. Feedback regulation of small RNA processing by the cleavage product. *RNA Biol* **16**: 1055-1065.

Gonzalez GM, Durica-Mitic S, Hardwick SW, Moncrieffe MC, Resch M, Neumann P, Ficner R, Görke B, Luisi BF. 2017. Structural insights into RapZ-mediated regulation of bacterial amino-sugar metabolism. *Nucleic Acids Res* **45**: 10845-10860.

Göpel Y, Khan MA, Görke B. 2016. Domain swapping between homologous bacterial small RNAs dissects processing and Hfq binding determinants and uncovers an aptamer for conditional RNase E cleavage. *Nucleic Acids Res* **44**: 824-837.

Göpel Y, Papenfort K, Reichenbach B, Vogel J, Görke B. 2013. Targeted decay of a regulatory small RNA by an adaptor protein for RNase E and counteraction by an anti-adaptor RNA. *Genes Dev* **27**: 552-564.

Guzman LM, Belin D, Carson MJ, Beckwith J. 1995. Tight regulation, modulation, and high-level expression by vectors containing the arabinose P_BAD_ promoter. *J Bacteriol* **177**: 4121-4130.

Kalamorz F, Reichenbach B, März W, Rak B, Görke B. 2007. Feedback control of glucosamine-6-phosphate synthase GlmS expression depends on the small RNA GlmZ and involves the novel protein YhbJ in *Escherichia coli*. *Mol Microbiol* **65**: 1518-1533.

Karimova G, Pidoux J, Ullmann A, Ladant D. 1998. A bacterial two-hybrid system based on a reconstituted signal transduction pathway. *Proc Natl Acad Sci U S A* **95**: 5752-5756.

Keseler IM, Mackie A, Santos-Zavaleta A, Billington R, Bonavides-Martinez C, Caspi R, Fulcher C, Gama-Castro S, Kothari A, Krummenacker M et al. 2017. The EcoCyc database: reflecting new knowledge about *Escherichia coli* K-12. *Nucleic Acids Res* **45**: D543-D550.

Love MI, Huber W, Anders S. 2014. Moderated estimation of fold change and dispersion for RNA-seq data with DESeq2. *Genome Biol* **15**: 550.

Lüttmann D, Göpel Y, Görke B. 2012. The phosphotransferase protein EIIA^Ntr^ modulates the phosphate starvation response through interaction with histidine kinase PhoR in *Escherichia coli*. *Mol Microbiol* **86**: 96-110.

Lüttmann D, Heermann R, Zimmer B, Hillmann A, Rampp IS, Jung K, Görke B. 2009. Stimulation of the potassium sensor KdpD kinase activity by interaction with the phosphotransferase protein IIA^Ntr^ in *Escherichia coli*. *Mol Microbiol* **72**: 978-994.

Morita T, Kawamoto H, Mizota T, Inada T, Aiba H. 2004. Enolase in the RNA degradosome plays a crucial role in the rapid decay of glucose transporter mRNA in the response to phosphosugar stress in *Escherichia coli*. *Mol Microbiol* **54**: 1063-1075.

Otto C, Stadler PF, Hoffmann S. 2014. Lacking alignments? The next-generation sequencing mapper segemehl revisited. *Bioinformatics* **30**: 1837-1843.

Quinlan AR, Hall IM. 2010. BEDTools: a flexible suite of utilities for comparing genomic features. *Bioinformatics* **26**: 841-842.

Schnetz K, Stülke J, Gertz S, Krüger S, Krieg M, Hecker M, Rak B. 1996. LicT, a *Bacillus subtilis* transcriptional antiterminator protein of the BglG family. *J Bacteriol* **178**: 1971-1979.

Wolfinger MT, Fallmann J, Eggenhofer F, Amman F. 2015. ViennaNGS: A toolbox for building efficient next- generation sequencing analysis pipelines. *F1000Research* **4**: 50.
